# Catalytic Mechanism of ATP Hydrolysis in the ATPase Domain of Human DNA Topoisomerase IIα

**DOI:** 10.1021/acs.jcim.2c00303

**Published:** 2022-08-10

**Authors:** Mitja Ogrizek, Matej Janežič, Katja Valjavec, Andrej Perdih

**Affiliations:** † National Institute of Chemistry, Hajdrihova 19, SI-1001 Ljubljana, Slovenia; ‡ Faculty of Pharmacy, University of Ljubljana, Aškerčeva 7, SI 1000 Ljubljana, Slovenia

## Abstract

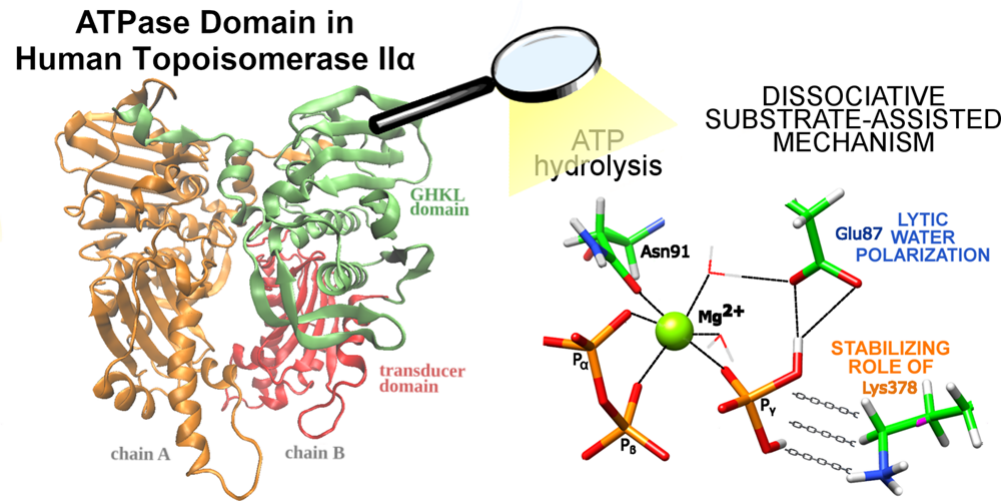

Human DNA topoisomerase IIα is a biological nanomachine that regulates the topological changes of the DNA molecule and is considered a prime target for anticancer drugs. Despite intensive research, many atomic details about its mechanism of action remain unknown. We investigated the ATPase domain, a segment of the human DNA topoisomerase IIα, using all-atom molecular simulations, multiscale quantum mechanics/molecular mechanics (QM/MM) calculations, and a point mutation study. The results suggested that the binding of ATP affects the overall dynamics of the ATPase dimer. Reaction modeling revealed that ATP hydrolysis favors the dissociative substrate-assisted reaction mechanism with the catalytic Glu87 serving to properly position and polarize the lytic water molecule. The point mutation study complemented our computational results, demonstrating that Lys378, part of the important QTK loop, acts as a stabilizing residue. The work aims to pave the way to a deeper understanding of these important molecular motors and to advance the development of new therapeutics.

## 1 Introduction

DNA topoisomerases encompass a family of complex biological nanomachines that can catalyze the induction of topological changes in the DNA molecules.^1,2^ By modulating DNA topology, they play an essential role in several crucial processes in the cell such as transcription, replication, and chromosome segregation.^3−5^ They are divided into two groups: type I topoisomerases, which cleave a single strand of the double-stranded DNA, and type II topoisomerases, which cleave both strands of the DNA.^6,7^ Both types of DNA topoisomerases are essential for DNA replication and transcription in all living cells as they maintain steady-state distributions of topological states.^8^

**Figure 1:**
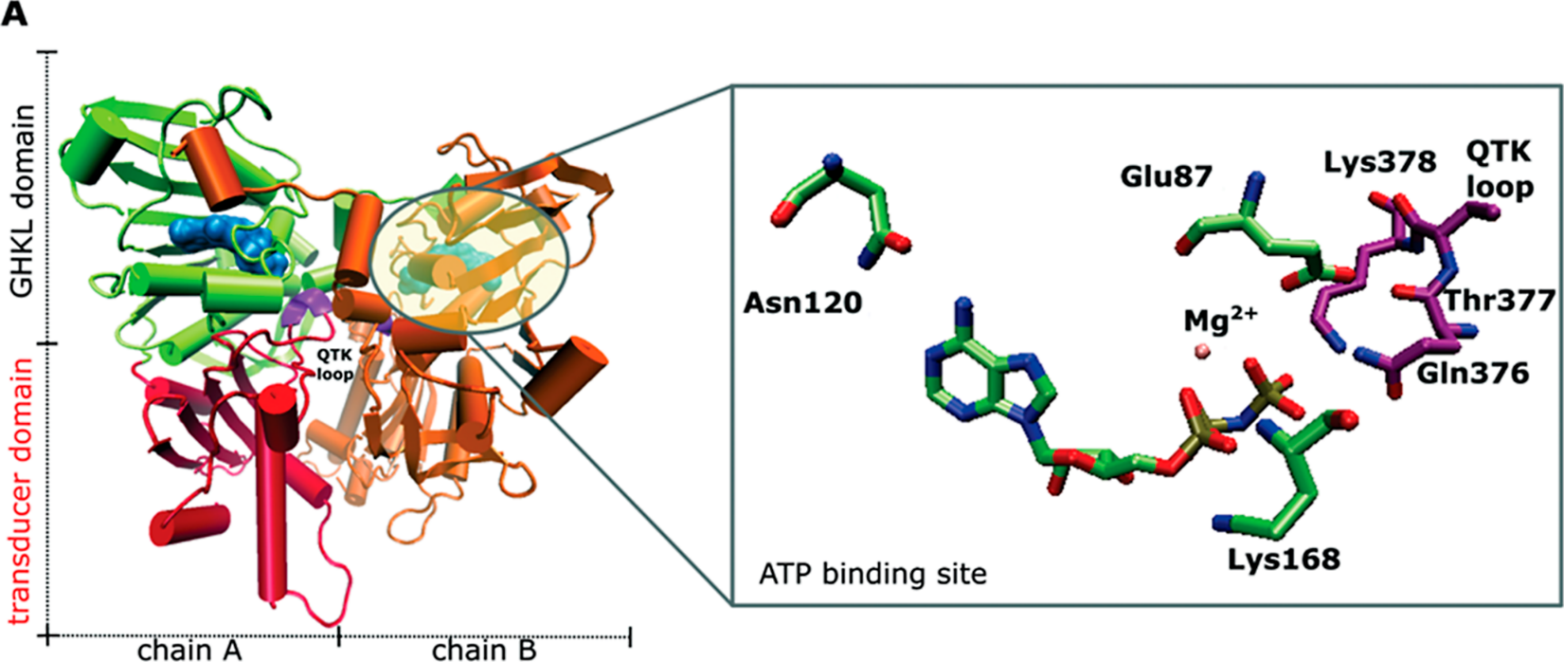
Human topoisomerase IIα ATPase dimer (PDB: 1ZXM), (Left): Green—GHKL domain of chain A, red—transducer domain of chain A, orange—chain B, violet—QTK loop, and blue—ATP. Right: Zoom in on the ATP binding site with the bound AMP–PNP ligand.

The human DNA topoisomerase IIα (htIIα) is a stable homodimer protein^9^ and its structure can be divided into three separate domains.^10^ The N-terminal or the ATPase domain is a part of the GHKL (Gyrase, Hsp90, histidinKinase, MutL) family of proteins and binds the T-segment of the DNA (Figure 1).^11,12^ The central domain binds and cleaves the DNA G-segment. Finally, the C-terminal section contains residues important for phosphorylation as well as interactions with other proteins.^6,7^ The enzyme also undergoes acetylation, with several possible modification sites on the ATPase and central domains; in particular, the eukaryotic-specific Lys168 in the ATP binding pocket was found to be key for the dimerization of the ATPase domain.^13^ The proposed catalytic cycle of a type II topoisomerase is characterized by a two-gate mechanism, where the homodimer shows remarkable flexibility as the molecular gates undergo substantial opening and closing motions.^14−17^

The role of the ATP molecule in type II topoisomerases has been a subject of intensive research.^14,18−21^ Biochemical studies revealed that type II topoisomerases hydrolyze two ATP molecules per reaction cycle. The role of the first ATP molecule is assumed to support a unidirectional transfer of the T-segment of DNA from its binding at the N-terminal domain to its exit via the C-gate of the enzyme after the G-segment is cleaved at the DNA gate.^22^ The role of the second ATP hydrolysis is even more ambiguous and some research suggests to be linked with the communication between the ATPase and the cleavage/ligation domains to support catalysis by resetting the enzyme conformation.^23^

The studies of the reaction mechanism of ATP hydrolysis in type II topoisomerases show that after binding of two ATP molecules, the enzyme stochastically hydrolyzes the first ATP before hydrolyzing the second ATP molecule. More precisely, following the hydrolysis of the first ATP, at least one slow step in the mechanism occurs before products of the second ATP hydrolysis can be detected. The sequential hydrolysis of ATP and product release steps indicate the complexity via which type II topoisomerase operate.^14^ In human topoisomerase IIα, it was further shown that only one ATP molecule is required for the enzyme to fully complete its catalytic cycle, while nonhydrolyzable ATP analogues allow the enzyme to complete only a single turnover of DNA transport and leave it unable to reset for a new cycle.^14,23^ ATP hydrolysis and ADP dissociation are important for rapid kinetics and opening of the N-gate, allowing the enzyme to enter into a succeeding catalytic cycle.^14,24^ Biochemical investigations also studied the role of the QTK loop (376QTK378), which extends from its transducer domain to the ATP-binding pocket in the GHKL domain, and concluded it to be an important feature for interdomain communication (Figure 1). It keeps the N-terminal gate open in the absence of nucleotides and facilitates the N-gate dimerization once a nucleotide binds. Deletion causes increased baseline cleavage levels but sharply reduces strand passage and virtually abolishes stimulation of cleavage by nucleotides. Furthermore, while the low baseline ATPase activity is retained, DNA stimulation of ATPase activity is abolished. Lastly, the operation of the N-gate becomes disordered; it can trap DNA not only in the presence of non-hydrolysable ATP analogues but also in the absence of nucleotides.^25^

Many structural studies investigated the mechanism of type II topoisomerases and predominantly focused on their DNA-binding/cleavage core of the enzyme.^26−28^ The ATPase domain of htIIα was also studied separately, revealing a rigid-body movement of the structural modules within this domain.^29^ Recently, an important breakthrough occurred with the determination of two conformations of htIIα bound to DNA trapped by the topo II poison etoposide. The study provided valuable information on how the ATPase domain is spatially connected to the DNA-binding/cleavage domain conformations and that the bound ATP analogue enables the rotation of the N-gate and opening of the DNA gate.^10^

The ATPase domain of htIIα is also a promising new target site for an emerging class of potential anticancer drugs, called catalytic topo II inhibitors.^30,31^ These molecules include a class that targets the ATP binding site of htIIα and predominantly mimics the adenine moiety of ATP, with a variety of scaffolds already discovered and validated.^32−35^ Such inhibition of topo II would, in principle, not result in an induction of excessive DNA damage, characterized by the DNA double strand breaks that are one of the main triggers of severe adverse effects observed when administering topo II poisons (e.g., etoposide and doxorubicin). These harmful effects comprise incidences of secondary malignancies after chemotherapy and cardiotoxicity.^36−38^ Furthermore, the increased occurrence drug resistance to clinically used topo II poisons further fuels the need for revisiting this established anticancer target.^39^

With the aim to unravel some of the intricacies linked with the mechanism of action type II topoisomerases, we focused on the ATPase dimer of htIIα, and employed all-atom molecular simulations, multiscale quantum mechanics (QM)/molecular mechanics (MM) reaction modeling, and a point mutation study. The collected data offer new atomistic insights into this fascinating biological nanomachine and could support the development of new cancer therapeutics targeting this domain.

## 2 Results and Discussion

### 2.1 Dynamics of Human Topo IIα ATPase Dimer

To study the dynamics of the of the ATPase domain dimer and assess the influence of the ATP ligand, we performed molecular dynamics (MD) simulations of the ATPase topo II dimer with bound ATP (holo system), and its apo form where both ATPs were removed. Initially, we assessed the metrics connected with the overall system stability during the simulations. RMSD values for the apo dimer and the system with bound ATP were 2.9 and 3.4 Å, respectively. Visualizing the trajectories, we observed that, the elevated RMSD in the holo structure can be attributed to the opening of the transducer domain, which is not as prevalent in the apo structure. Interestingly, the resulting conformation of the holo system resembles the solved topoisomerase II-ADP ATPase dimer complex structure (PDB: 1ZXN); that is, the likely structure after hydrolysis of the first ATP molecule has occurred.^29^ (Supporting Information, Sections S1 and S2).

The bound ATP molecules were stable in their binding sites with the RMSD of about 0.8 Å in both protomers. This was further reflected by the stability of other bonds than anchor the ATP to its binding pocket. The bond between the amide oxygen of Asn120 and the amino group of the ATP averaged 3 Å. Similarly, the average distance of the H-bond between the side chain nitrogen of Lys168 and the oxygen on the α-phosphate was stable at 2.7 Å. The Mg^2+^ ion also showed little spatial movement; RMSD was 0.5 Å. Additionally, we measured the interactions observed in the X-ray structure between the Mg^2+^ ion and its interacting partners—ATP, water molecules, and relevant amino acid residues determining that they all remained stable and within the observed distances completing the octahedral coordination of this ion (Table S1). With the crude system stability established, we proceeded to focus on catalysis-relevant parts of the htIIα ATPase domain in more detail.

First, we examined the Glu87 residue that was shown to be an essential residue for the catalysis.^40^ In the crystal structure, Glu87 interacts with a water molecule that is already optimally positioned for a nucleophilic attack on the γ-phosphate of the ATP. During the simulation, the candidate for the catalytic water molecule was firmly nested in the ATP binding site and present throughout the simulation. The catalytic Glu87 also interacted with the ATP molecule. We monitored the bond lengths between the side chain oxygens of the Glu87 carboxylic group and the oxygen on the γ-phosphates. We noticed that in chain A, the carboxylic side chain group of Glu87 exhibits rotations, alternating which of its oxygen atoms was closer (∼3.5 Å) and which farther (∼5.5 Å) from the γ-phosphate, whereas in chain B, no such rotation was observed (Figure S2B). This marks the only substantial difference observed between chains A and B during our simulation. Next, we examined the QTK loop, which is completely conserved^41^ and is critical for the interdomain communication and potentially also plays a role in the ATP catalytic reaction.^25^ In both the holo and the apo system, the loop remains stable in both chains/monomers of the ATPase domain throughout the simulation (RMSD = 2 Å and 1.6 Å). Additional graphs for all analyzed distances and RMSD values can be found in Section S2 of the Supporting Information.

**Figure 2:**
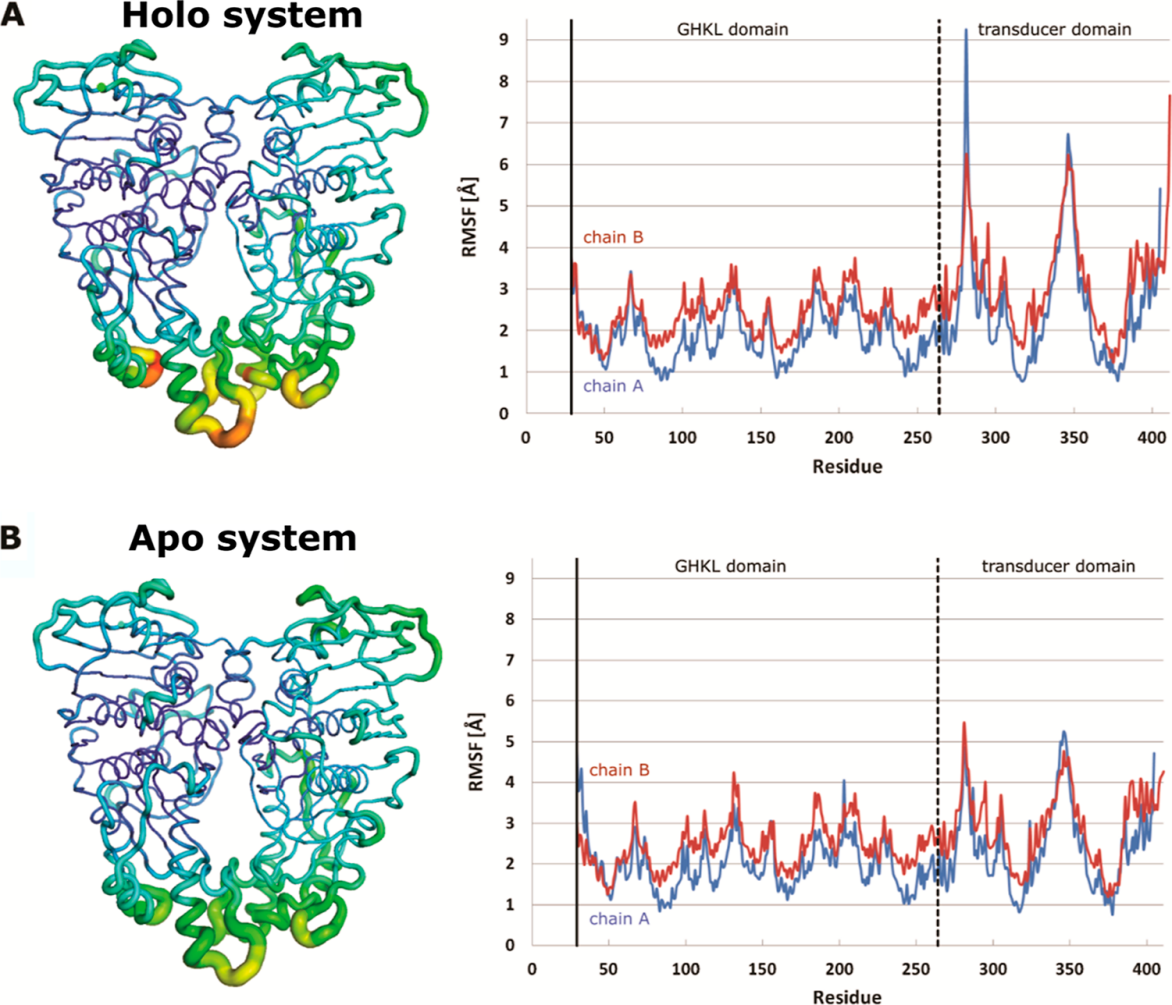
RMSF heatmaps and per residue RMSF graphs of the simulated htIIα dimers: (A) the holo system and (B) the apo system without ATP. Blue on the graph represents the A chain and red the B chain of the ATPase dimer. Our model is missing the first 28 residues. The dotted line shows a divide between the GHKL domain (residues 29–264) and the transducer domain (residues 265–428).

To further quantify the observed dimer dynamics, we performed a principal component analysis (PCA) and calculated per-residue root mean square fluctuation (RMSF) values. The RMSF protein heatmaps in Figure 2 again reflect a higher flexibility of the transducer domain versus the GHKL domain. The different levels of movement between the GHKL and the transducer domain are also evident on the per-residue RMSF graphs. It is interesting to note that the apo system exhibited less mobility in the transducer domain and that after 100 ns, the holo system was closer to the structure of 1ZXN, that is, the supposed structure of topo IIα after hydrolysis has occurred (see Section S1).

**Figure 3:**
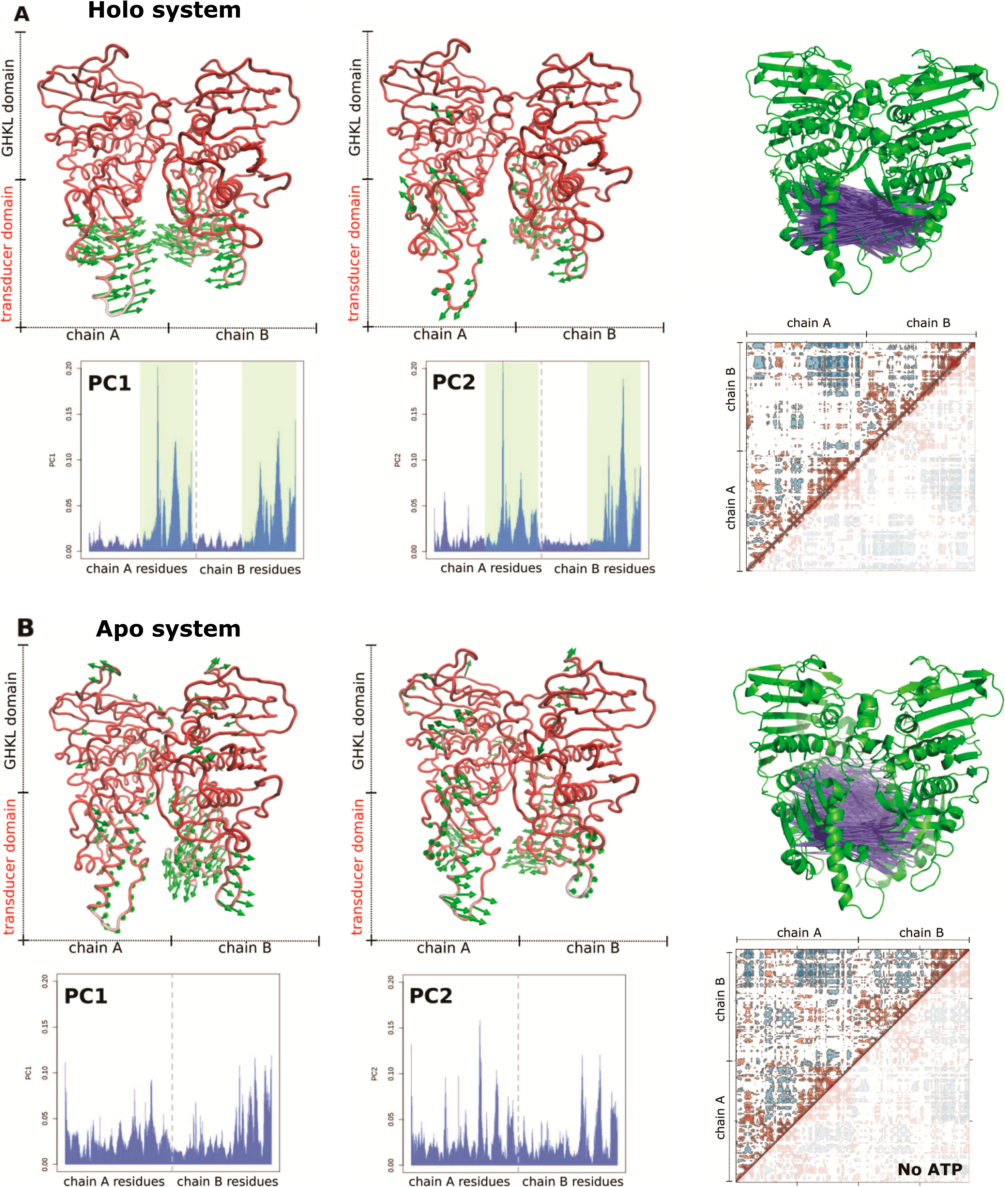
(Left) Eigenvectors and per-residue contribution for the first and second principal component for the (A) holo system and (B) apo system. On graphs the dotted line shows a divide between chain A and B. The GHKL domain (residues 29–264) is unshaded, and the transducer domain (residues 265–428) is shaded in green for the holo system. (Right) Residual correlative motions visualized by the dynamic cross-correlation maps for the following: (A) holo system and (B) apo system. The color scale in the matrices goes from blue (for values ranging between −1 and −0.25), through white (−0.25 to 0.25) to red (0.25 to 1). Negative values depict residue pairs having the opposite motion, namely, anti-correlated motions, while positive values indicate pairwise movement in the same direction. Above each matrix, the three-dimensional cartoon model shows the strongest anti-correlated motions in each structure (i.e., blue lines connecting the residue pairs).

In PCA for the holo system, the obtained eigenvectors of the first principal component (PC1) indicate, as expected, the opening/closing of the transducer domain. The vectors of the second principal component (PC2) are almost perpendicular to the first, signifying a twisting motion (Figure 3A). The system without ATP is less ordered in its movements, reflected both in the vector and per residue graphs (Figure 3B) and (Animation 1). This is in agreement with the observation that ATP binding promotes concerted movement of the ATPase domain.^18^

We also determined the correlation of the displacements of all residue pairs and plotted the correlations in the dynamic cross-correlation matrices (DCCM) outlined in Figure 3. With DCCM analysis, we wanted to confirm and additionally characterize the domain dynamics previously studied in PCA. Already at first glance, we can detect differences in the degree of (anti)correlated motions that each system exhibits. Comparing the two matrices of the htIIα dimers, we can observe a presence of clearer patterns in the holo system, whereas the apo system exhibits no larger concentrated groups of either positively or negatively correlated motions. Visualizing the strongest anti-correlations in three-dimensional representation confirmed our observations, as we can detect a clearer cluster of anti-correlations in the holo transducer domain compared to the apo system. All results support the notion that binding of the ATP influences the dynamics of the ATPase dimer.

It should be noted that our truncated topo IIα systems lack the DNA molecule, the absence of which likely influences global protein shifts. Therefore, the conformational motions of the protein cannot be fully captured within the current simulations. More accurate evaluation of the dynamics would require a more comprehensive conformational space sampling of the system^42^ and the inclusion of other components of the topo II molecular motor. Therefore, these simulations serve only as an initial assessment of the dimer’s dynamics.

### 2.2 Reaction Mechanism of ATP Hydrolysis

Next, we investigated the catalytic process of ATP hydrolysis on a single ATPase domain. Molecular modeling of such processes requires the application of quantum mechanics.^43,44^ A standard multiscale QM/MM approach involves dividing the system into a reaction region described by QM, while the remaining part is treated with classical MM.^45,46^ The determination of the course of events in the transformation from the initial (reactants) to the final (products) configuration of the system is a task solved by the reaction pathway methods.^47^ One group of methods comprises the restrained coordinate driving (RCD)^47^ where the reaction is forced to occur by restraining the system to a predefined chemically relevant reaction coordinate. Unfortunately, RCD methods suffer from hysteresis difficulties due to the constraints imposed on the reaction coordinate.^48^ Another approach to determine the reaction pathways that can circumvent this concern is the replica path method (RPATh).^48−51^ It is an extension of the self-penalty walk methods,^52,53^ which involves a simultaneous optimization of a set of geometries of the system corresponding to a set of points along the reaction path, resulting in an approximate minimum energy pathway (MEP). RPATh removes the bias in choosing the reaction coordinate because the method uses the root mean square distance (RMSD) parameter to define the distance between two points on the approximate MEP. This allows the “global pathway” movement to define a reaction path rather than a combination of distances used by RCD.^54−56^

The literature lists two major mechanisms regarding the ATP hydrolysis.^57^ One is a general base catalysis (GBC) where the catalytic base abstracts a proton from the lytic water molecule prior to or at the transition state (TS). An alternative to GBC is the substrate-assisted catalysis (SAC), in which the γ-phosphate group of the ATP molecule acts as the catalytic base for the cleavage of the lytic water.^58,59^ The mechanism of ATP hydrolysis has been computationally investigated in several biological systems, including ATP-dependent molecular motors such as myosin, F1-ATPase, and kinesin, proposing diverse reaction paths, especially concerning the lytic water proton transfer.^60−63^

**Figure 4:**
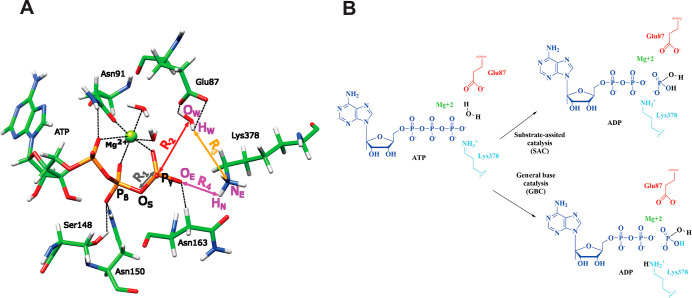
(A) ATP binding site on the htIIα and bound ATP molecule generated from the experimentally present AMP–PNP analogue (PDB: 1ZXM). Marked are also distances that we monitored during the modeling of the reaction pathways. (B) Schematics of the SAC and GBC reaction paths of ATP hydrolysis that were investigated in this study.

Again, the co-crystal structure of the htIIα ATPase domain with bound AMP–PNP offers an excellent modeling starting point as an approximation of the ATPase state before the hydrolysis takes place (Figure 4A). It further provides an optimal experimentally determined position of the lytic water molecule for the nucleophilic attack. In our study, we evaluated the GBC and SAC reaction mechanisms of ATP hydrolysis (Figure 4B). For modeling the QM region, we chose a well-established DFT hybrid functional B3LYP in conjunction with a standard 6-31G* basis set. Such a methodological setup has been shown to work well in previous computational studies of the catalytic mechanisms of ATP hydrolysis of various molecular motors.^62^

The QM region included three water molecules near the γ-phosphate, the Mg^2+^ ion, the methyl triphosphate fragment of ATP, and Glu87 and Lys378, that is, atoms/side chains possibly involved in H^+^ and OH^–^ transfer (Figure S5). Lys378 was posited as a catalytic base and modeled as protonated, as Lys residues near phosphates have been shown to be protonated, with deprotonation only occurring in highly apolar (hydrophobic) environments.^64−66^ We also examined potential roles of Gln376, which is also a part of the QTK loop. Considering the position of the side chain of this residue in relation to other partners, we could construct no feasible way for this residue to participate directly in proton transfer, for example, via a proton wire. Therefore, Gln376 was left in the region described by classic molecular mechanics, where its H-bonding effects on stabilizing ATP could still be fully included in the calculations.

**Figure 5:**
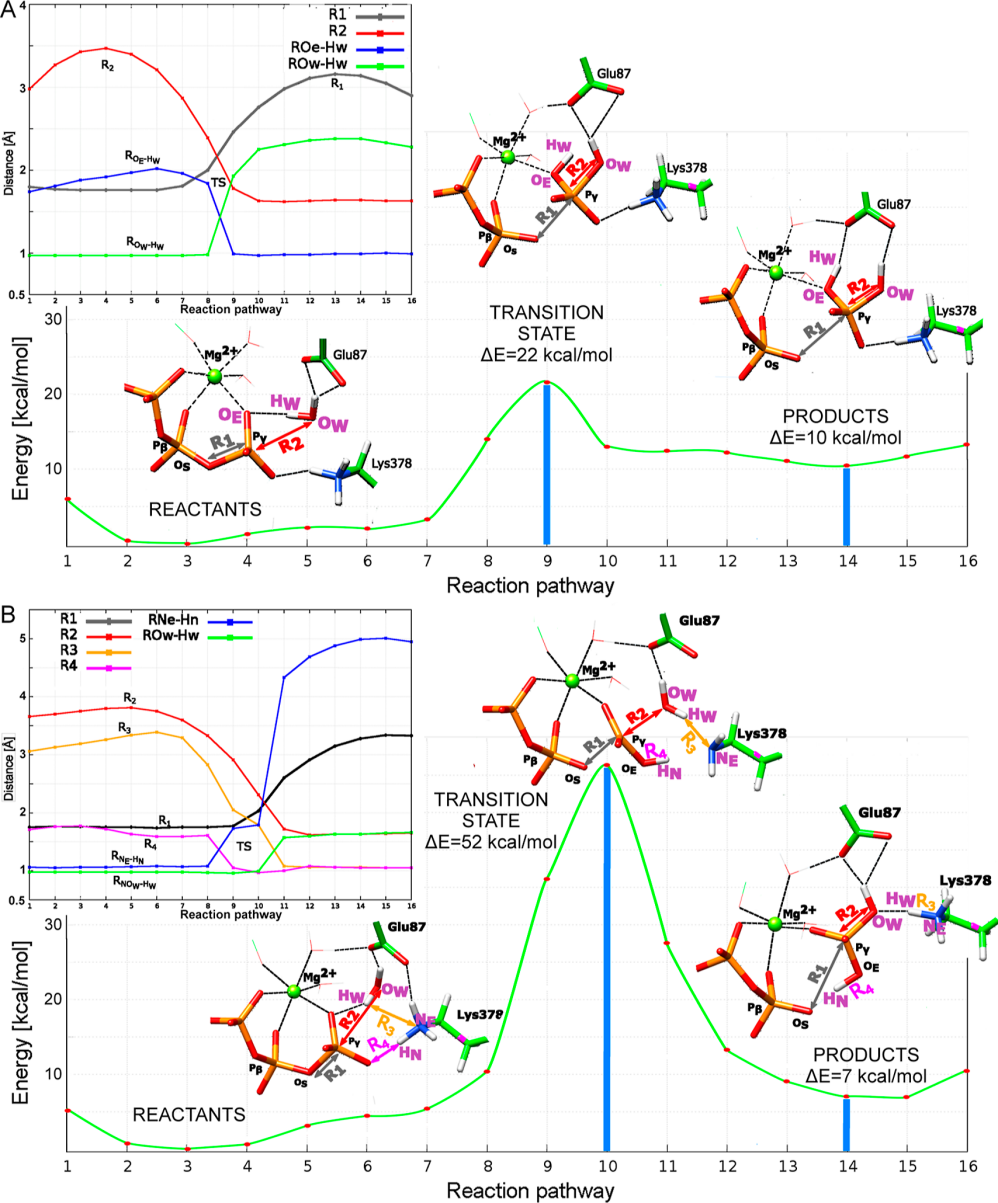
RPATh-determined reaction pathways with monitored key distances and reaction frames/replicas. Dashed lines denote coordination bonds with Mg^2+^ (green sphere) or formed hydrogen bonds. Distances are labeled in angstroms (Å). (A) SAC with key replicas 2, 9, and 16 of the reaction pathway and monitored *R*_1_, *R*_2_, *R*_O_e_–H_w_,_ and *R*_O_w_–H_w__ distances. (B) GBC with key replicas 4, 10, and 16 of the reaction pathway and monitored *R*_1_, *R*_2_, *R*_3_, *R*_4_, *R*_N_e_–H_n__, and *R*_O_w_–H_w__ distances.

In the obtained SAC reaction mechanism, the energy progression, changes of key distances, and key frames/replicas as determined by the RPATh method are depicted in Figure 5A (Animation 2). We defined four key distances to describe the reaction course. The *R*_1_ distance represents the breakage of the bond between the oxygen on β-phosphate (P_β_) and γ-phosphate (P_γ_), *R*_2_ monitors the transfer of the OH^–^ from the lytic water to the γ-phosphate. Finally, to describe the proton transfer of the H_w_ atom from the O_W_ oxygen of the lytic water to the O_E_ γ-phosphate oxygen, we measured *R*_O_e_–H_w__ and *R*_O_w_–H_w__ distances.

Between replicas 1 and 4, the lytic water molecule stabilized by two hydrogen bonds with Glu87 residue readjusted itself. Until step 4, the *R*_1_ distance remained consistent (1.75 vs 1.76 Å), while the *R*_2_ increased from 2.98 to 3.47 Å. Then, the system moved toward the TS and the P_γ_–O_S_ bond breaks in replica 8 (*R*_1_ = 1.94 Å). The γ-phosphate, O_S_, and water O_w_ oxygen atoms form a trigonal bipyramidal structure. The dissociation of the P_γ_–O_S_ bond thus occurs before the proton and OH^–^ transfer. In reaction step 9, OH^–^ and H_w_^+^ comprising the lytic water have already moved to O_E_ and P_γ_ atoms of ATP via the concerted reaction mechanism (*R*_O_e_–H_w__ = 0.99 Å, *R*_O_w_–H_w__ = 1.93 Å). In the succeeding replicas, a product state is reached (PS) in which *R*_1_ = 2.90 Å and *R*_2_ = 1.63 Å. The resulting energy profile has a structure approximating the TS with an activation energy of about 22.0 kcal/mol, which is reasonably close to previous theoretical and experimental studies of comparable molecular motors.^62^

Subsequently, we explored the GBC reaction mechanism of ATP hydrolysis. For this reaction mechanism, we measured six key distances to describe the reaction events and plotted them in Figure 5B (Animation 3). The *R*_1_ distance characterizes the breakage of the bond between the oxygen on β-phosphate and P_γ_. *R*_2_ monitors transfer of the OH^–^ nucleophile from the lytic water to the P_γ_. *R*_3_ and O_w_–H_w_ monitor the transfer of H_w_ from the lytic water to Lys378. With *R*_4_ and N_E_–H_N_ distances, we follow the proton transfer from the QTK Lys378 to the γ-phosphate species.

In the first four reactions steps, the system slightly readjusts itself; *R*_1_ and *R*_4_ remained stable, while *R*_2_ increases from 3.66 to 3.80 Å, and *R*_3_ from 3.06 to 3.26 Å. Then, the system shifts toward the TS. In step 9, the proton transfers from the N_E_ nitrogen of Lys378 to the O_E_ of the γ-phosphate, signified by the *R*_N_e_–H_n__ increasing to 1.73 Å and *R*_4_ decreasing to 1.05 Å. ATP and the lytic water stabilized via the interaction with Glu87 continue to reorient themselves in replica 10. The lytic water approaches both P_γ_ and N_E_, causing a decrease in *R*_2_ and *R*_3_ to 2.31 Å and 1.79 Å, respectively. In this replica, *R*_1_ reaches 2.03 Å and the phosphodiester bond breaks. We again find P_γ_ forming a trigonal bipyramidal TS together with O_S_ and O_w_ atoms. In the next reaction step, there is a simultaneous transfer of lytic water’s H_w_^+^ proton and OH^–^ nucleophile to the N_E_ atom on Lys378 and γ-phosphorus of ATP. The products state is finally reached in the succeeding replicas with *R*_1_ distance of 3.33 Å and *R*_2_ of 1.65 Å (replica 16). With the activation energy of 52 kcal/mol, the GBC reaction mechanism is energetically much less favorable compared to the pathway obtained for SAC.

Comparing the energy of the products, the GBC configuration displays lower energy. The observed higher energy of the product structure in the SAC is probably related to the final position of the proton transferred from the lytic water molecule to the oxygen of the γ-phosphate. Unlike in GBC, it does not enable the formation of a H-bond between the γ-phosphate and ADP. This suggests that in SAC, some additional readjusting of the hydrolyzed γ-phosphate is likely to occur to lead to more favorable product energy. Although not presently modeled, such a readjustment would not change the main feature—the dissociative reaction mechanism.

In both investigated variations of the ATP hydrolysis, we observed a dissociative reaction mechanism with a simultaneous H_w_^+^ proton, and OH^–^ transfer of the lytic water in and could not identify a TS with only the OH^–^ species being present. Such a dissociative mechanism has already been previously observed in multiscale simulations of other relevant biomolecular systems^60^ including molecular motors,^62,67−69^ which depend on the chemical energy of ATP to execute motility processes. Observed TS formed by the P_γ_, O_S_, and O_w_ atoms resembled a trigonal bipyramidal structure. Our computational results heavily favored SAC and indeed, the need for a GB could be considered ambiguous, as the catalytic benefit of the water deprotonation is estimated to be rather meagre.^58^

Reaction pathways also showed that both Glu87 and Lys378 favorably stabilize the TS but have a different role in the catalytic reaction. Lys378 primarily stabilizes the reactant state and TS via H-bonding with the non-bridging oxygen of the γ-phosphate, rather than interacting with Glu87 or with the lytic water molecule. For the Glu87 residue, earlier studies, on htIIα and DNA gyrase pinned it as a catalytic residue.^40,70^ During our study, we performed GBC calculations with Glu87 acting as a catalytic base, but our attempts were unsuccessful as no stable structure with a protonated Glu87 could be obtained. Instead our QM/MM simulations suggested Glu87 plays its catalytic role by properly positioning the crucial lytic water molecule. This would enable substantial catalysis of the reaction rendering the successful attack of P_γ_ possible.^58^ In addition, simulations of ATP hydrolysis in other molecular motors already established the polarization of involved waters by a glutamate residue.^62^

Performed multiscale simulations of ATP hydrolysis catalyzed by molecular motors often suggested that the reaction takes place via a proton-wire reaction mechanism involving uncoordinated water molecules facilitating the lytic H^+^ transfer. In the active site of htIIα, there are two waters in the vicinity of the P_γ_, atom that could theoretically support a similar proton transfer mechanism via further involvement of the Asp94 residue. However, both of these water molecules are tightly coordinated with the Mg^2+^ ion and remained so during the multiscale reaction modeling where they were included in the QM region. As mentioned, molecular simulations also demonstrated that the interaction between Mg^2+^ and these water molecules is stable. All this taken into consideration makes the wire transfer mechanism an unlikely catalytic strategy for the htIIα system.

The used QM DFT setup worked well in our case; nevertheless, it should be noted that the use of newly developed DFT functionals such as M06-2X and basis sets might provide even superior description of the studied reaction. Finally, the application of the RPATh method allowed the generation of an approximate MEP that is not biased toward a prechosen reaction coordinate. However, a more detailed representation of the reaction free energy surface would require more advanced methods with more comprehensive sampling. This could be achieved, for example, by metadynamics simulations^71^ or QM/MM methods based on free energy perturbations to provide more precise insights into the reaction energetics and potentially reinvestigate and reassess the steps and roles in the reaction mechanism.

### 2.3 Experimental Study of the Lys378 Role

To further verify the role of Lys378, we sought to experimentally characterize ATP hydrolysis in a K378A htIIα mutant. In all expression experiments, the cells appeared to be infected by appearance. However, the protein appeared to be expressed at quite low levels or not at all on the sodium dodecyl sulfate-polyacrylamide gel electrophoresis gels and in the western blots. The latter showed that some intact protein was present 24 h after infection, with some degradation products also visible. After 48 h, no intact protein was seen. This suggests that the mutant is degraded upon expression, and we confirmed this by western blot analyzes (see Supporting Information, Section S3). Interestingly, when the analogous Lys337 residue in bacterial DNA gyrase was mutated to Glu, stability problems also occurred; activity began to slowly decline over time.^72^ The mutant protein behaved as expected during purification in a larger scale infection, that is, it behaved as the wild-type (WT) protein However, the yield was still low because the amount of protein present in the starting material was low. It did not appear to degrade during the purification process. The final protein concentration of the K378A htIIα mutant was 25 μg/mL. The K378A mutant was initially tested for its activity in the topo II-mediated decatenation assay and was found to possess activity (data not shown). The WT used as a control was at 200 μg/mL.

**Figure 6:**
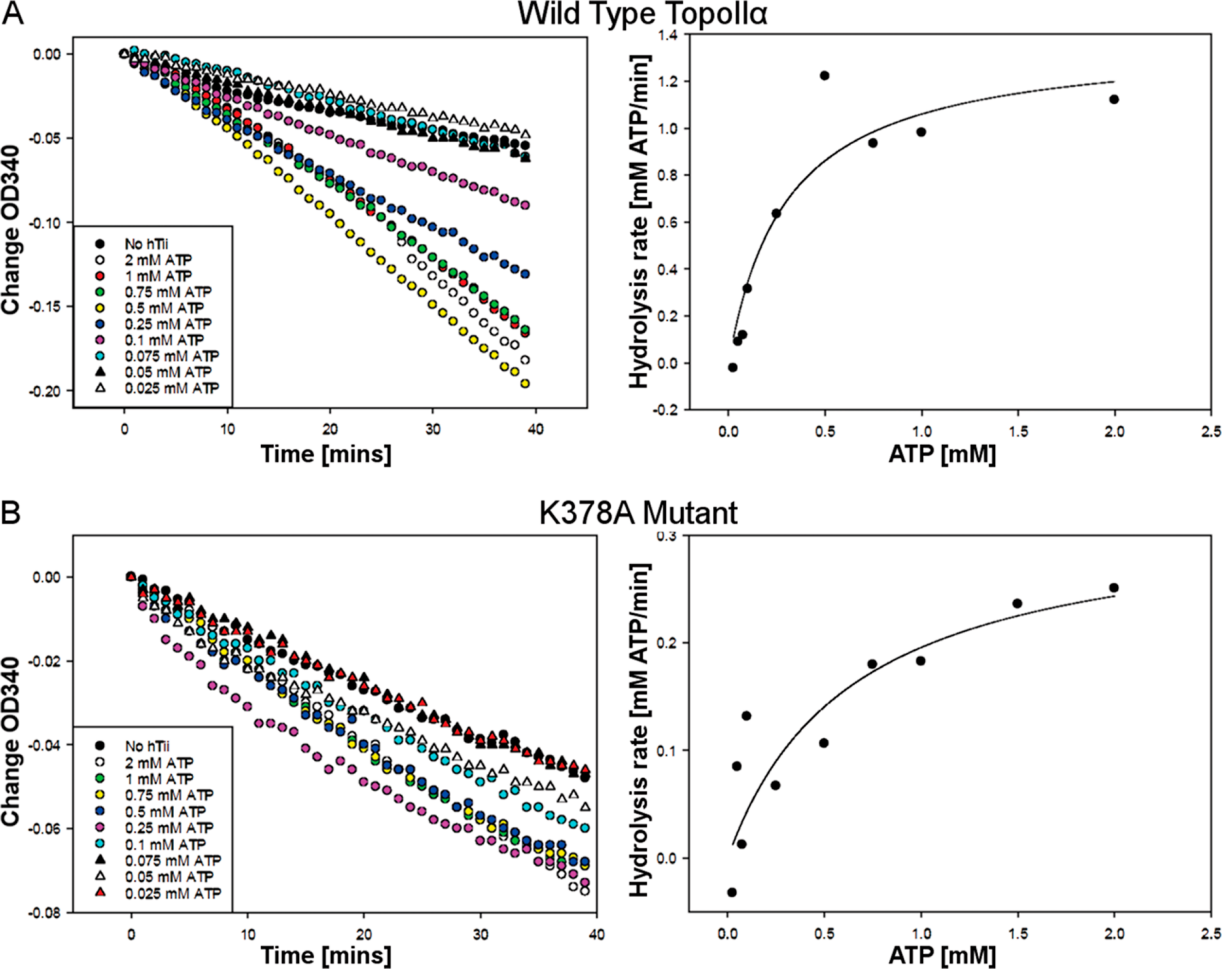
Results of the ATPase assay: (A) WT htIIα and (B) K378A mutant. The rates were plotted against the ATP concentration and curves fitted using the hyperbolic equation *y* = *ax*/(*b* + *x*), where *y* = rate, *x* = [ATP], *a* = *V*_max_, and *b* = *K*_m_. Detailed data in Section S4 of the Supporting Information.

Having verified that the K378A htIIα mutant retained its function via the decatenation study, we moved to examine the impact of this point mutation on the ATPase activity. The data from the runs after the addition of the ATP were plotted and lines were then fitted to this data by a linear regression (Figure 6). The rates for each ATP concentration were then calculated as μM ATP hydrolyzed/min and rates for both WT and K378A htIIα mutant were then plotted against the ATP concentration in Figure 6 and curves were fitted to the obtained data using a hyperbolic equation. Individual and average calculated values are further depicted in Table 1.

**Table 1:** ATPase Assay; Calculated *K*_m_ (mM ATP) and *V*_max_ (μM ATP/min) Values

	WT run 1	WT run 2	av. WT	K378 run 1	K378 run 2	K378 run 3	av. K378
*V*_max_ (μM/min)	1.22	0.96	1.09	0.23	0.29	0.25	0.26
*K*_m_ (mM)	0.24	0.22	0.23	0.15	0.12	0.096	0.122
*k*_cat_ (s^–1^)	1.27	1.00	1.14	0.55	0.69	0.6	0.65

In the K378A mutant, the *K*_m_ and *k*_cat_ were reduced to about half of the WT htIIα, while the *V*_max_ was a mere 25% of the original. When the analogous Lys337 was mutated to Glu in bacterial DNA gyrase B, the loss of ATPase activity was much more profound. The mutant domain had a 10^3^-fold decrease in kcat, and for the full-length enzyme, ATPase rates were more than 10 times lower.^72^

The results of the performed point-mutation study nicely complemented and substantiated our multiscale computational results of reaction modeling. Lys378 is not essential for the catalysis, and the most likely mechanism of ATP hydrolysis in htIIα is SAC. However, Lys378 does seem to play an important role in enzymatic efficiency. We deduce it helps in the stabilization of the reactants and the TS so that the ATP hydrolysis can proceed smoothly; in the reaction profile, we observed stable H-bonding of the side chain of Lys378 with the non-bridging oxygen of the γ-phosphate of the ATP. Additionally, mutations of the analogous Lys359 in *Drosophila* showed dramatically reduced but not completely abolished ATPase activity, severe reduction of the stimulating effect of DNA on ATP hydrolysis, and also disruption of the N-gate operating mechanism; the enzyme could trap DNA even without ATP or ATP analogues present.^73^ Same was true for a mutant of htIIα with a deleted QTK loop.^25^ This positions the QTK loop as essential for interdomain communication and Lys378 as having the additional role of a sensor amino acid required for the correct operation of the clamp. Broadly, the proposed role of Lys378 is consistent with observations in several molecular motors, such as myosin, kinesin, and F1-ATPase, in which a stabilizing contribution has been attributed to the Lys residue located near the transferred γ-phosphate of ATP.^62^ Moreover, such a stabilizing function appears to be observed in other systems in which phosphate group transfer occurs,^74−76^ further confirming the proposed catalytic mechanism in this biomolecular system.

## 3 Conclusions

Human DNA topoisomerase IIα is a biological nanomachine that regulates DNA topological changes. It is also an established anticancer target that is currently being revisited in the development of new catalytic inhibitors, some of which target the ATP binding site. To further unravel some of the mechanistic aspects of the htIIα mode of action, we have combined all-atom simulations, multiscale QM/MM calculations, and a point mutation study to investigate the htIIα ATPase domain dimer. All-atom simulations of the holo and apo systems of the htIIα ATPase domain dimer showed that the binding of ATP influences its dynamics. Furthermore, using multiscale QM/MM methods, we investigated two reaction mechanisms of ATP hydrolysis, substrate-assisted and GBC. The computational results indicated that ATP hydrolysis proceeds via a dissociative mechanism, consistent with the proposed pattern observed when studying ATP-driven molecular motors, and heavily favored the SAC reaction pathway. To supplement our computational results and further asses the probability of the two reaction mechanisms, we constructed a K378A htIIα point mutant, which revealed that Lys378 plays a role in stabilizing the reacting complex but does not act as a catalytic base. The obtained results thus positioned the determined SAC mechanism of ATP hydrolysis as more probable. The catalytic Glu87 residue was not observed to act as a catalytic base, but instead served to properly position and polarize the lytic water molecule, which is also consistent with previous studies. We hope that this work will contribute to a deeper understanding of these intriguing molecular motors and provide new information to advance the development of new therapeutics.

## 4 Experimental Section

### 4.1 All-Atom Simulations of the Human Topo ΙΙα Dimer

The X-ray structure of ATPase domain dimer of htIIα with bound non-hydrolysable ATP analogues AMP–PNP is publicly available (PDB: 1ZXM). MD calculations were performed using the CHARMM molecular modeling suite.^77^ The missing side chains and protein loop residues not provided in the 1ZXM PDB file were generated using the PDB Hydro web server (http://lorentz.dynstr.pasteur.fr/pdb/index.php) in a two-stage procedure.^78^ First, the 1ZXM crystal structure was submitted to the PDB Hydro web server to construct the missing protein loop. The server automatically detected the missing loop residues and added them into the structure.^79^ The upgraded structure was retrieved and visually inspected to assess the generated conformation of the loop. Another module of the Hydro server was then used to detect the missing side chain and screen possible rotamers of these side chains selecting those with the lowest van der Waals energy to be added to the structure. The process was done consecutively for each residue resulting in the final structure with all protein atoms included.^78^ Also, the AMP–PNP ligand present in the 1ZXM structure was changed to ATP. CHARMM-GUI^80^ was used to generate the solvated complexes and two systems of the human topo IIα dimer were subsequently prepared for MD simulations, (1) htIIα devoid of ATP ligands (apo structure) and (2) a topo II dimer with two ATP and two Mg^2+^ ions bound to their corresponding binding sites (holo structure).

CHARMM parameter and topology files (version 26) specified the force field parameters of the amino acid residues of the topo II ATPase dimers.^81,82^ CHARMM general force field (CGenFF) modeled the partial charges and atom types of the ATP molecule.^83^ The system was immersed into TIP3 water molecules^84^ with truncated octahedral shape and edge distance of 10 Å. Chlorine and potassium ions were added to make the system electroneutral with further ions added to the final KCl concentration of 0.15 M. Ion placement was performed using the Monte Carlo method. The periodic boundary conditions were applied and grid information for the particle-mesh Ewald fast Fourier transform was generated automatically. The apo and holo topo II structures comprised 78,948 and 79,078 atoms, respectively. Short energy minimizations were then carried out to remove bad contacts. Both topo II systems were subsequently minimized by the steepest descent method, followed by the modified adopted basis Newton–Raphson (ABNR) method (both for 2000 steps) and an equilibration MD of 4 ns using 1 fs simulation step. The production MD trajectories were generated by leap-frog integration scheme and 2 fs simulation step coupled with SHAKE algorithm. A 0.10 μs long MD simulation production runs were performed for each structure. Sampling of conformations occurred every 100th step extracting 50,000 conformations for analysis. Visualization and analysis of the geometry parameters of the production MD trajectories were performed using Visual MD (VMD) program.^85^

#### 4.1.1 Analysis of the MD Trajectories

MD Trajectories were examined by employing the Cpptaj module of AmberTools 18^86^ to calculate root mean square deviations (RMSDs), RMSFs, and PCA. Bio3D library (version 2.3-0)^87^ within the R environment enabled the calculations of the dynamic cross-correlation maps (DCCMs). Results were visualized by VMD,^85^ R software environment^88^ and PyMOL.^89^

##### 4.1.1.1 Principal Component Analysis

Covariance matrices of positional fluctuations were built and diagonalized to obtain the eigenvalues, and their corresponding eigenvectors. The eigenvectors possessing the largest eigenvalues correspond to the most relevant motions sampled in the simulation (known as principal components, PCs). We calculated the cumulative variances accounted by the PCs for both systems. First two PCs were considered in further explanation of differences in motions between simulations. In the holo system and apo system, first two PCs captured approximately 40% of overall position fluctuations. We used the normal mode Wizard^90^ plugin of VMD program to visualize the large-scale collective motions observed in our simulations.

##### 4.1.1.2 Dynamical Cross-Correlation Map (DCCM) Calculations

The cross-correlation maps were calculated using the DCCM function in the Bio3D package, which determines the covariance matrices and computes the Pearson correlation coefficient (*C*_*ij*_) on the Cα atom pairs, *i* and *j* via eq 1
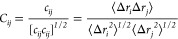
1

In the equation, *c*_*ij*_ is defined as , and Δ*r*_*i*_ represents a displacement vector of atom *i* and Δ*r*_*j*_ of atom *j* with the brackets signifying an ensemble average. Cross-correlation coefficient *C*_*ij*_ was obtained by normalizing the covariances. *C*_*ij*_ values range from −1 to 1, corresponding to type of correlated motion between pairs. The positive vales indicate positively correlated motions (movement in the same direction), while the negative values indicate the negatively correlated motions, anticorrelations (movement in the opposite direction). Visualization of the anticorrelated motions corresponding to the residues in the matrices was completed in PyMOL.

### 4.2 Multiscale QM/MM Study of the ATP Hydrolysis

#### 4.2.1 Preparation of the Reactant and Product Structures

Again, we used coordinates of one protomer of the experimentally determined ATPase dimer of the htIIα complexed with the bound AMP–PNP ligand (PDB: 1ZXM). We left crystal waters inside the system as they were considered a part of the reaction mechanism. Multiscale QM/MM calculations^46^ were also performed within the CHARMM environment.^91^ HBUILD command was used to add hydrogen atoms. Then, the system was solvated with a TIP3P waters cubic box, and 20 potassium and 13 chloride ions were added as counterions, making the system neutral. The topo II system was divided to the QM region which was treated with quantum mechanics, while the rest of the system was described with molecular mechanics. The QM region encompassed the methyl-triphosphate fragment of the ATP molecule, lytic water, Mg^2+^ ion, two Mg^2+^-coordinated water molecules, and side chains of residues Lys378 and Glu87, that is, atoms that could feasibly take part in the H^+^ and OH^–^ transfer (Figure S5). For the MM part of the calculations, we used CHARMM (version 36). QM DFT calculations were performed using GAMESS (general atomic and molecular electronic structure system),^92^ interfaced with CHARMM. The quantum regions were treated with the B3LYP DFT method and 6-31G* basis set. ABNR minimization algorithm was used for geometry optimization. MM calculations were performed with a dielectric constant of ε = 1 with a classical force shift method and a cutoff distance of 12 Å. The empty valances of the QM Glu87, Lys378, and ATP moieties were filled with a link atoms (hydrogen).

We used CHARMM restrain distance (RESD) methodology (eq 2) to form required covalent bonds of the product structures where the ATP hydrolysis was completed. This was achieved by adding restrains to the CHARMM energy function, thereby directing the movement along the chemically relevant reaction coordinate and forcing the breakage/formation of the anticipated chemical bond (water hydrolysis, proton transfer).^47^
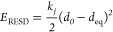
2*k*_*j*_ denotes an applied force constant, *d*_0_ is the current distance between a pair of selected atoms, and *d*_eq_ is the targeted/equilibrium distance.

For the SAC mechanism, the product structure was obtained by first restraining a distance between the γ-phosphorus atom P_γ_ and the O_w_ of the lytic water molecule to 1.6 Å in 100 steps of RESD QM/MM minimization and then minimizing the obtained structure with the previous constraint removed until the root-mean-squared gradient was smaller than 0.0001 kcal/mol/Å. For the product structure resulting from the GBC reaction mechanism, a similar procedure was applied only more restrains were introduced. Here, we restrained (1) the γ-phosphorus atom P_γ_ and the O_w_ of the lytic water molecule to 1.6 Å, (2) the H_w_ proton of the lytic molecule and N_E_ of Lys378 to 1 Å, and (3) the proton from the N_E_ of Lys378 and O_E_ oxygen of P_γ_ to 1 Å. The obtained structure was then minimized without the present restrains.

#### 4.2.2 Generation of Reaction Pathways

The replica path (RPATh) method^49^ is an extension of the self-penalty walk methods that can model reaction pathways.^52,53^ The 16 initial replicas of both investigated reaction pathways were created by a linear interpolation of coordinates between atoms of the QM/MM minimized starting and the corresponding product structures. The method further uses a combined minimization involving the sum of the configurational energies and two RPATh penalty energy terms. First penalty term restrains (eq 3) the distances between adjacent replica points. This safeguards that the pathway is regularly spaced and smooth.
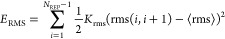
3

*N*_REP_ is the number of generated replicas and rms(*i*,*i* + 1) is the best fit RMS value (eq 4) between successive replicas
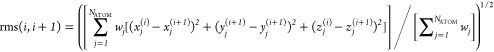
4⟨rms⟩ denotes the average RMS value (eq 5), while *w*_*j*_ is the atom weighting factor (eq 6)
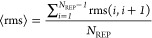
5
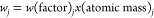
6

To restrain the angle between an adjacent and the next adjacent pathway points, we used a second RPATh force constant, which uses the law of cosines, and with that, we avoid paths that double back on themselves (eqs 7–9). In this manner, the RPATh method ensures that the optimized replicas represent the reaction pathway.^48,49^
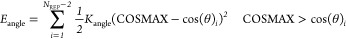
7
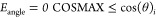
8
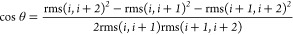
9

QM/MM RPATh optimizations of the initially generated two pathways were performed by applying an ABNR method.^50^ Parameters of the RPATh penalty terms were as follows: *K*_rms_ = 2000.0 kcal/mol/Å^2^, *K*_angle_ = 100.0 kcal/mol/Å, and COSMAX = 0.95 radian. To evade the overestimation of contributions of those atoms not involved in the reaction, only the QM region was weighted in the RPATh RMS calculation.^50^ Pathways were optimized for 10,000 steps or until for at least 30 consecutive steps, the total pathway root mean square gradient was less than 0.01 kcal/mol/Å and the change in total pathway energy was less than 1.0 kcal/mol. After initial reaction pathways were obtained, we further optimized them by selecting the new initial and end coordinates closer to the observed TS. We then recalculated the pathways by generating again 16 replicas using the same settings for RPATh penalty terms. This process could be subject to multiple iterations to obtain appropriate initial and end reaction coordinates. Finally, the energy profiles were calculated for both reaction mechanisms under investigation.

### 4.3 Construction and Expression of the Human Topo IIα K378A Mutant

The K378A mutant was made in the WT human htIIα gene in the pFastbac vector. This was used to transform DH10bac cells and positive clones were identified by sequencing of the mutant gene. The bacmid was used to transfect *Spodoptera frugiperda* (SF21) insect cells. Virus from the transfection was used to infect more insect cells to amplify the virus stock. A larger (300 mL) culture of insect cells was infected with the P4 stock of virus, grown 24 h and the human topo IIα purified by nuclear preparation, followed by column chromatography. Expression of the protein was confirmed by western blot using polyclonal anti-human topo II alpha antibodies.

### 4.4 ATPase Assay of the WT and htIIα K378A Mutant

The ATPase activity of both systems was examined using a linked assay. The hydrolysis of ATP produces ADP which coupled with pyruvate kinase/lactate dehydrogenase mix leads to a conversion of NADH into NAD. The reduction of NADH is then monitored at 340 nm. A mix of assay buffer (5 μL of 10× buffer per assay: final conc. 20 mM Tris-HCl, 5 mM magnesium acetate, 125 mM potassium acetate, 2 mM dithiothreitol, pH = 7.9), linear pBR322 (1.5 μL of 1 mg/mL per assay), phosphoenol pyruvate (0.5 μL of 80 mM per assay), pyruvate kinase/lactate dehydrogenase mix (0.75 μL per assay), NADH (1 μL of 20 mM per assay), dimethyl sulfoxide (1 μL per assay), and water (32.85 μL per assay) was first prepared. 41.1 μL was aliquoted into the wells of a 384-well microtiter plate. 5 μL of the dilution buffer or human topo II (WT; 170 nM stock giving 17 nM final concentration: K378; 70 nM stock giving 7 nM final) was then added and mixed. The OD340 was monitored for 10 min and then the reaction started by adding 3.4 μL of the fitting concentration of ATP and the OD340 was monitored for up to 40 min. Assays were performed at 37 °C and two negative controls (dilution buffer but no enzyme) were also run.

## 5 Data and Software Availability

All molecular simulations, analysis, and visualization were performed with widely used programs available freely for academic institutions: CHARMM (version 40), GAMESS (May 2013 R1 release), AmberTools 18, PyMol 2.0, VMD 1.9.2, and R software environment with the Bio3D library 2.3-0. The starting structure was obtained from the Protein Data Bank public structure database. All procedures and workflows are described in the Methods section. Structures used for MD and QM/MM replica path simulations in pdb format are provided in the Supporting Information.

## Supporting Information Available

Movement of the topo IIα ATPase transducer domain during the MD simulations, analysis of MD simulations, western blot to check for the expression of K378 topo IIα mutant, results of the ATPase assay, and 2D scheme of the QM region (PDF)
First principal component in the apo and holo systems (MP4)
SAC reaction mechanism (MP4)
GBC reaction mechanism (MP4)
Structure files (ZIP)

## References

[ref1] Bates A. D.; Maxwell A. DNA topology: Topoisomerases keep it simple. Curr. Biol. 1997, 7, R778–R781. 10.1016/s0960-9822(06)00403-9. 9382831

[ref2] Nitiss J. L. DNA topoisomerase II and its growing repertoire of biological functions. Nat. Rev. Cancer 2009, 9, 327–337. 10.1038/nrc2608. 19377505 PMC2730144

[ref3] Nitiss J. L. Investigating the biological functions of DNA topoisomerases in eukaryotic cells. Biochim. Biophys. Acta, Gene Struct. Expression 1998, 1400, 63–81. 10.1016/s0167-4781(98)00128-6. 9748506

[ref4] Wang J. C. Cellular roles of DNA topoisomerases: A molecular perspective. Nat. Rev. Mol. Cell Biol. 2002, 3, 430–440. 10.1038/nrm831. 12042765

[ref5] Pommier Y.; Sun Y.; Huang S. N.; Nitiss J. L. Roles of eukaryotic topoisomerases in transcription, replication and genomic stability. Nat. Rev. Mol. Cell Biol. 2016, 17, 703–721. 10.1038/nrm.2016.111. 27649880 PMC9248348

[ref6] Champoux J. J. DNA topoisomerases: Structure, function, and mechanism. Annu. Rev. Biochem. 2001, 70, 369–413. 10.1146/annurev.biochem.70.1.369. 11395412

[ref7] Schoeffler A. J.; Berger J. M. DNA topoisomerases: harnessing and constraining energy to govern chromosome topology. Q. Rev. Biophys. 2008, 41, 41–101. 10.1017/s003358350800468x. 18755053

[ref8] Vos S. M.; Tretter E. M.; Schmidt B. H.; Berger J. M. All tangled up: how cells direct, manage and exploit topoisomerase function. Nat. Rev. Mol. Cell Biol. 2011, 12, 827–841. 10.1038/nrm3228. 22108601 PMC4351964

[ref9] Tennyson R. B.; Lindsley J. E. Type II DNA topoisomerase from Saccharomyces cerevisiae is a stable dimer. Biochemistry 1997, 36, 6107–6114. 10.1021/bi970152f. 9166781

[ref10] Vanden Broeck A.; Lotz C.; Drillien R.; Haas L.; Bedez C.; Lamour V. Structural basis for allosteric regulation of Human Topoisomerase IIα. Nat. Commun. 2021, 12, 2962 10.1038/s41467-021-23136-6. 34016969 PMC8137924

[ref11] Chène P. ATPases as drug targets: Learning from their structure. Nat. Rev. Drug Discovery 2002, 1, 665–673. 10.1038/nrd894. 12209147

[ref12] Dutta R.; Inouye M. GHKL, an emergent ATPase/kinase superfamily. Trends Biochem. Sci. 2000, 25, 24–28. 10.1016/s0968-0004(99)01503-0. 10637609

[ref13] Bedez C.; Lotz C.; Batisse C.; Broeck A. V.; Stote R. H.; Howard E.; Pradeau-Aubreton K.; Ruff M.; Lamour V. Post-translational modifications in DNA topoisomerase 2α highlight the role of a eukaryote-specific residue in the ATPase domain. Sci. Rep. 2018, 8, 9272 10.1038/s41598-018-27606-8. 29915179 PMC6006247

[ref14] Baird C. L.; Harkins T. T.; Morris S. K.; Lindsley J. E. Topoisomerase II drives DNA transport by hydrolyzing one ATP. Proc. Natl. Acad. Sci. U.S.A. 1999, 96, 13685–13690. 10.1073/pnas.96.24.13685. 10570133 PMC24125

[ref15] Roca J.; Berger J. M.; Harrison S. C.; Wang J. C. DNA transport by a type II topoisomerase: Direct evidence for a two-gate mechanism. Proc. Natl. Acad. Sci. U.S.A. 1996, 93, 4057–4062. 10.1073/pnas.93.9.4057. 8633016 PMC39486

[ref16] Roca J.; Wang J. C. DNA transport by a type II DNA topoisomerase: evidence in favor of a two-gate mechanism. Cell 1994, 77, 609–616. 10.1016/0092-8674(94)90222-4. 8187179

[ref17] Lee S.; Jung S.-R.; Heo K.; Byl J. A. W.; Deweese J. E.; Osheroff N.; Hohng S. DNA cleavage and opening reactions of human topoisomerase IIα are regulated via Mg^2+^-mediated dynamic bending of gate-DNA. Proc. Natl. Acad. Sci. U.S.A. 2012, 109, 2925–2930. 10.1073/pnas.1115704109. 22323612 PMC3286967

[ref18] Bates A. D.; Berger J. M.; Maxwell A. The ancestral role of ATP hydrolysis in type II topoisomerases: prevention of DNA double-strand breaks. Nucleic Acids Res. 2011, 39, 6327–6339. 10.1093/nar/gkr258. 21525132 PMC3159449

[ref19] Bates A. D.; Maxwell A. The role of ATP in the reactions of type II DNA topoisomerases. Biochem. Soc. Trans. 2010, 38, 438–442. 10.1042/bst0380438. 20298198

[ref20] Bates A. D.; Maxwell A. Energy coupling in type II topoisomerases: Why do they hydrolyze ATP?. Biochemistry 2007, 46, 7929–7941. 10.1021/bi700789g. 17580973

[ref21] Stuchinskaya T.; Mitchenall L. A.; Schoeffler A. J.; Corbett K. D.; Berger J. M.; Bates A. D.; Maxwell A. How Do Type II Topoisomerases Use ATP Hydrolysis to Simplify DNA Topology beyond Equilibrium? Investigating the Relaxation Reaction of Nonsupercoiling Type II Topoisomerases. J. Mol. Biol. 2009, 385, 1397–1408. 10.1016/j.jmb.2008.11.056. 19094994 PMC4343537

[ref22] Hauk G.; Berger J. M. The role of ATP-dependent machines in regulating genome topology. Curr. Opin. Struct. Biol. 2016, 36, 85–96. 10.1016/j.sbi.2016.01.006. 26827284 PMC4785063

[ref23] Skouboe C.; Bjergbaek L.; Oestergaard V. H.; Larsen M. K.; Knudsen B. R.; Andersen A. H. A Human Topoisomerase IIα Heterodimer with Only One ATP Binding Site Can Go through Successive Catalytic Cycles. J. Biol. Chem. 2003, 278, 5768–5774. 10.1074/jbc.m210332200. 12480934

[ref24] Harkins T. T.; Lindsley J. E. Pre-steady-state analysis of ATP hydrolysis by Saccharomyces cerevisiae DNA topoisomerase II. 1. A DNA-dependent burst in ATP hydrolysis. Biochemistry 1998, 37, 7292–7298. 10.1021/bi9729099. 9585543

[ref25] Bendsen S.; Oestergaard V. H.; Skouboe C.; Brinch M.; Knudsen B. R.; Andersen A. H. The QTK Loop Is Essential for the Communication between the N-Terminal ATPase Domain and the Central Cleavage–Ligation Region in Human Topoisomerase IIα. Biochemistry 2009, 48, 6508–6515. 10.1021/bi9005978. 19485418

[ref26] Schmidt B. H.; Burgin A. B.; Deweese J. E.; Osheroff N.; Berger J. M. A novel and unified two-metal mechanism for DNA cleavage by type II and IA topoisomerases. Nature 2010, 465, 641–644. 10.1038/nature08974. 20485342 PMC2882514

[ref27] Dong K. C.; Berger J. M. Structural basis for gate-DNA recognition and bending by type IIA topoisomerases. Nature 2007, 450, 1201–1205. 10.1038/nature06396. 18097402

[ref28] Chen S. F.; Huang N. L.; Lin J. H.; Wu C. C.; Wang Y. R.; Yu Y. J.; Gilson M. K.; Chan N. L. Structural insights into the gating of DNA passage by the topoisomerase II DNA-gate. Nat. Commun. 2018, 9, 3085 10.1038/s41467-018-05406-y. 30082834 PMC6078968

[ref29] Wei H.; Ruthenburg A. J.; Bechis S. K.; Verdine G. L. Nucleotide-dependent domain movement in the ATPase domain of a human type IIA DNA topoisomerase. J. Biol. Chem. 2005, 280, 37041–37047. 10.1074/jbc.m506520200. 16100112

[ref30] Nitiss J. L. Targeting DNA topoisomerase II in cancer chemotherapy. Nat. Rev. Cancer 2009, 9, 338–350. 10.1038/nrc2607. 19377506 PMC2748742

[ref31] Pogorelcnik B.; Perdih A.; Solmajer T. Recent Advances in the Development of Catalytic Inhibitors of Human DNA Topoisomerase IIα As Novel Anticancer Agents. Curr. Med. Chem. 2013, 20, 694–709. 10.2174/092986713804999402. 23210851

[ref32] Pogorelčnik B.; Brvar M.; Žegura B.; Filipič M.; Solmajer T.; Perdih A. Discovery of mono- and disubstituted 1H-pyrazolo[3,4]pyrimidines and 9H-purines as catalytic inhibitors of human DNA topoisomerase IIα. ChemMedChem 2015, 10, 345–59. 10.1002/cmdc.201402459. 25522133

[ref33] Loboda K. B.; Valjavec K.; Štampar M.; Wolber G.; Žegura B.; Filipič M.; Dolenc M. S.; Perdih A. Design and synthesis of 3,5-substituted 1,2,4-oxadiazoles as catalytic inhibitors of human DNA topoisomerase IIα. Bioorg. Chem. 2020, 99, 103828 10.1016/j.bioorg.2020.103828. 32315896

[ref34] Bergant K.; Janežič M.; Valjavec K.; Sosič I.; Pajk S.; Štampar M.; Žegura B.; Gobec S.; Filipič M.; Perdih A. Structure-guided optimization of 4,6-substituted-1,3,5-triazin-2(1H)-ones as catalytic inhibitors of human DNA topoisomerase IIα. Eur. J. Med. Chem. 2019, 175, 330–348. 10.1016/j.ejmech.2019.04.055. 31096154

[ref35] Bergant Loboda K.; Janežič M.; Štampar M.; Žegura B.; Filipič M.; Perdih A. Substituted 4,5′-Bithiazoles as Catalytic Inhibitors of Human DNA Topoisomerase IIα. J. Chem. Inf. Model. 2020, 60, 3662–3678. 10.1021/acs.jcim.0c00202. 32484690 PMC7469689

[ref36] Bailly C. Contemporary challenges in the design of topoisomerase II inhibitors for cancer chemotherapy. Chem. Rev. 2012, 112, 3611–3640. 10.1021/cr200325f. 22397403

[ref37] Minotti G.; Menna P.; Salvatorelli E.; Cairo G.; Gianni L. Anthracyclines: Molecular advances and pharmacologic developments in antitumor activity and cardiotoxicity. Pharmacol. Rev. 2004, 56, 185–229. 10.1124/pr.56.2.6. 15169927

[ref38] Felix C. A. Secondary leukemias induced by topoisomerase-targeted drugs. Biochim. Biophys. Acta 1998, 1400, 233–255. 10.1016/s0167-4781(98)00139-0. 9748598

[ref39] Pilati P.; Nitti D.; Mocellin S. Cancer resistance to type II topoisomerase inhibitors. Curr. Med. Chem. 2012, 19, 3900–3906. 10.2174/092986712802002473. 22788766

[ref40] Gardiner L. P.; Roper D. I.; Hammonds T. R.; Maxwell A. The N-Terminal Domain of Human Topoisomerase IIα Is a DNA-Dependent ATPase. Biochemistry 1998, 37, 16997–17004. 10.1021/bi9818321. 9836594

[ref41] Caron P. R.; Wang J. C. Appendix II: Alignment of Primary Sequences of DNA Topoisomerases. Adv. Pharmacol. 1994, 29B, 271–297. 10.1016/s1054-3589(08)61143-6. 8996613

[ref42] Knapp B.; Ospina L.; Deane C. M. Avoiding False Positive Conclusions in Molecular Simulation: The Importance of Replicas. J. Chem. Theory Comput. 2018, 14, 6127–6138. 10.1021/acs.jctc.8b00391. 30354113

[ref43] Cavalli A.; Carloni P.; Recanatini M. Target-related applications of first principles quantum chemical methods in drug design. Chem. Rev. 2006, 106, 3497–3519. 10.1021/cr050579p. 16967914

[ref44] Warshel A.; Levitt M. Theoretical studies of enzymic reactions: Dielectric, electrostatic and steric stabilization of the carbonium ion in the reaction of lysozyme. J. Mol. Biol. 1976, 103, 227–249. 10.1016/0022-2836(76)90311-9. 985660

[ref45] Field M. J.; Bash P. A.; Karplus M. A combined quantum mechanical and molecular mechanical potential for molecular dynamics simulations. J. Comput. Chem. 1990, 11, 700–733. 10.1002/jcc.540110605.

[ref46] Eurenius K. P.; Chatfield D. C.; Brooks B. R.; Hodoscek M. Enzyme mechanisms with hybrid quantum and molecular mechanical potentials. I. Theoretical considerations. Int. J. Quantum Chem. 1996, 60, 1189–1200. 10.1002/(sici)1097-461x(1996)60:6<1189::aid-qua7>3.0.co;2-w.

[ref47] Monard G.; Prat-Resina X.; González-Lafont A.; Lluch J. M. Determination of enzymatic reaction pathways using QM/MM methods. Int. J. Quantum Chem. 2003, 93, 229–244. 10.1002/qua.10555.

[ref48] Woodcock H. L.; Hodošček M.; Sherwood P.; Lee Y. S.; Schaefer H. F.; Brooks B. R. Exploring the quantum mechanical/molecular mechanical replica path method: a pathway optimization of the chorismate to prephenate Claisen rearrangement catalyzed by chorismate mutase. Theor. Chem. Acc. 2003, 109, 140–148. 10.1007/s00214-002-0421-3.

[ref49] Woodcock H. L.; Hodošček M.; Gilbert A. T. B.; Gill P. M. W.; Schaefer H. F.; Brooks B. R. Interfacing Q-chem and CHARMM to perform QM/MM reaction path calculations. J. Comput. Chem. 2007, 28, 1485–1502. 10.1002/jcc.20587. 17334987

[ref50] Woodcock H. L.; Hodošček M.; Brooks B. R. Exploring SCC-DFTB paths for mapping QM/MM reaction mechanisms. J. Phys. Chem. A 2007, 111, 5720–5728. 10.1021/jp0714217. 17555303

[ref51] Lee Y. S.; Pike V. W.; Hodoscek M. Identification of the transition states in the inversion of 1,4-benzodiazepines with the ab initio replica path method. J. Phys. Chem. A 2008, 112, 1604–1611. 10.1021/jp077738o. 18229901

[ref52] Czerminski R.; Elber R. Reaction path study of conformational transitions in flexible systems: Applications to peptides. J. Chem. Phys. 1990, 92, 5580–5601. 10.1063/1.458491.

[ref53] Elber R.; Karplus M. A method for determining reaction paths in large molecules: Application to myoglobin. Chem. Phys. Lett. 1987, 139, 375–380. 10.1016/0009-2614(87)80576-6.

[ref54] Borišek J.; Pintar S.; Ogrizek M.; Turk D.; Perdih A.; Novic M. A Water-Assisted Catalytic Mechanism in Glycoside Hydrolases Demonstrated on the Staphylococcus aureus Autolysin E. ACS Catal. 2018, 8, 4334–4345. 10.1021/acscatal.8b01064.

[ref55] Perdih A.; Hodoscek M.; Solmajer T. MurD ligase fromE. coli: Tetrahedral intermediate formation study by hybrid quantum mechanical/molecular mechanical replica path method. Proteins: Struct., Funct., Bioinf. 2009, 74, 744–759. 10.1002/prot.22188. 18704940

[ref56] Sosič I.; Gobec M.; Brus B.; Knez D.; Živec M.; Konc J.; Lešnik S.; Ogrizek M.; Obreza A.; Žigon D.; Janežič D.; Mlinarič-Raščan I.; Gobec S. Nonpeptidic Selective Inhibitors of the Chymotrypsin-Like (β5i) Subunit of the Immunoproteasome. Angew. Chem., Int. Ed. Engl. 2016, 55, 5745–5748. 10.1002/anie.201600190. 27037901

[ref57] Admiraal S. J.; Herschlag D. Mapping the transition state for ATP hydrolysis: implications for enzymatic catalysis. Chem. Biol. 1995, 2, 729–739. 10.1016/1074-5521(95)90101-9. 9383480

[ref58] Lassila J. K.; Zalatan J. G.; Herschlag D. Biological phosphoryl-transfer reactions: understanding mechanism and catalysis. Annu. Rev. Biochem. 2011, 80, 669–702. 10.1146/annurev-biochem-060409-092741. 21513457 PMC3418923

[ref59] Allen K. N.; Dunaway-Mariano D. Phosphoryl group transfer: evolution of a catalytic scaffold. Trends Biochem. Sci. 2004, 29, 495–503. 10.1016/j.tibs.2004.07.008. 15337123

[ref60] Huang W.; Liao J. L. Catalytic Mechanism of the Maltose Transporter Hydrolyzing ATP. Biochemistry 2016, 55, 224–231. 10.1021/acs.biochem.5b00970. 26666844

[ref61] Wang C.; Huang W.; Liao J.-L. QM/MM Investigation of ATP Hydrolysis in Aqueous Solution. J. Phys. Chem. B 2015, 119, 3720–3726. 10.1021/jp512960e. 25658024

[ref62] Kiani F. A.; Fischer S. Comparing the catalytic strategy of ATP hydrolysis in biomolecular motors. Phys. Chem. Chem. Phys. 2016, 18, 20219–20233. 10.1039/c6cp01364c. 27296627

[ref63] Prieß M.; Göddeke H.; Groenhof G.; Schäfer L. V. Molecular Mechanism of ATP Hydrolysis in an ABC Transporter. ACS Cent. Sci. 2018, 4, 1334–1343. 10.1021/acscentsci.8b00369. 30410971 PMC6202651

[ref64] Richman D. E.; Majumdar A.; García-Moreno E E. Conformational Reorganization Coupled to the Ionization of Internal Lys Residues in Proteins. Biochemistry 2015, 54, 5888–5897. 10.1021/acs.biochem.5b00522. 26335188

[ref65] Kougentakis C. M.; Grasso E. M.; Robinson A. C.; Caro J. A.; Schlessman J. L.; Majumdar A.; García-Moreno E E. B. Anomalous Properties of Lys Residues Buried in the Hydrophobic Interior of a Protein Revealed with 15N-Detect NMR Spectroscopy. J. Phys. Chem. Lett. 2018, 9, 383–387. 10.1021/acs.jpclett.7b02668. 29266956

[ref66] Takayama Y.; Castañeda C. A.; Chimenti M.; García-Moreno B.; Iwahara J. Direct evidence for deprotonation of a lysine side chain buried in the hydrophobic core of a protein. J. Am. Chem. Soc. 2008, 130, 6714–6715. 10.1021/ja801731g. 18454523

[ref67] Schwarzl S. M.; Smith J. C.; Fischer S. Insights into the chemomechanical coupling of the myosin motor from simulation of its ATP hydrolysis mechanism. Biochemistry 2006, 45, 5830–5847. 10.1021/bi052433q. 16669626

[ref68] Dittrich M.; Hayashi S.; Schulten K. On the mechanism of ATP hydrolysis in F1-ATPase. Biophys. J. 2003, 85, 2253–2266. 10.1016/s0006-3495(03)74650-5. 14507690 PMC1303451

[ref69] Hayashi S.; Ueno H.; Shaikh A. R.; Umemura M.; Kamiya M.; Ito Y.; Ikeguchi M.; Komoriya Y.; Iino R.; Noji H. Molecular mechanism of ATP hydrolysis in F1-ATPase revealed by molecular simulations and single-molecule observations. J. Am. Chem. Soc. 2012, 134, 8447–8454. 10.1021/ja211027m. 22548707

[ref70] Jackson A. P.; Maxwell A. Identifying the catalytic residue of the ATPase reaction of DNA gyrase. Proc. Natl. Acad. Sci. U.S.A. 1993, 90, 11232–11236. 10.1073/pnas.90.23.11232. 8248233 PMC47956

[ref71] Barducci A.; Bonomi M.; Parrinello M. Metadynamics. Wiley Interdiscip. Rev.: Comput. Mol. Sci. 2011, 1, 826–843. 10.1002/wcms.31.

[ref72] Smith C. V.; Maxwell A. Identification of a residue involved in transition-state stabilization in the ATPase reaction of DNA gyrase. Biochemistry 1998, 37, 9658–9667. 10.1021/bi9801309. 9657678

[ref73] Hu T.; Chang S.; Hsieh T. S. Identifying Lys359 as a Critical Residue for the ATP-dependent Reactions of Drosophila DNA Topoisomerase II. J. Biol. Chem. 1998, 273, 9586–9592. 10.1074/jbc.273.16.9586. 9545289

[ref74] Murillo-López J.; Zinovjev K.; Pereira H.; Caniuguir A.; Garratt R.; Babul J.; Recabarren R.; Alzate-Morales J.; Caballero J.; Tuñón I.; Cabrera R. Studying the phosphoryl transfer mechanism of the E. coli phosphofructokinase-2: from X-ray structure to quantum mechanics/molecular mechanics simulations. Chem. Sci. 2019, 10, 2882–2892. 10.1039/c9sc00094a. 30996866 PMC6429617

[ref75] Mateeva T.; Klähn M.; Rosta E. Structural Dynamics and Catalytic Mechanism of ATP13A2 (PARK9) from Simulations. J. Phys. Chem. B 2021, 125, 11835–11847. 10.1021/acs.jpcb.1c05337. 34676749

[ref76] Recabarren R.; Osorio E. H.; Caballero J.; Tuñón I.; Alzate-Morales J. H. Mechanistic insights into the phosphoryl transfer reaction in cyclin-dependent kinase 2: A QM/MM study. PLoS One 2019, 14, e0215793 10.1371/journal.pone.0215793. 31483779 PMC6726203

[ref77] Brooks B. R.; Bruccoleri R. E.; Olafson B. D.; States D. J.; Swaminathan S.; Karplus M. CHARMM: A program for macromolecular energy, minimization, and dynamics calculations. J. Comput. Chem. 1983, 4, 187–217. 10.1002/jcc.540040211.

[ref78] Azuara C.; Lindahl E.; Koehl P.; Orland H.; Delarue M. PDB_Hydro: incorporating dipolar solvents with variable density in the Poisson-Boltzmann treatment of macromolecule electrostatics. Nucleic Acids Res. 2006, 34, W38–W42. 10.1093/nar/gkl072. 16845031 PMC1538897

[ref79] Hoffmann D.; Knapp E. W. Protein dynamics with off-lattice Monte Carlo moves. Phys. Rev. E: Stat. Phys., Plasmas, Fluids, Relat. Interdiscip. Top. 1996, 53, 4221–4224. 10.1103/physreve.53.4221. 9964743

[ref80] Jo S.; Kim T.; Iyer V. G.; Im W. CHARMM-GUI: A web-based graphical user interface for CHARMM. J. Comput. Chem. 2008, 29, 1859–1865. 10.1002/jcc.20945. 18351591

[ref81] MacKerell A. D.; Bashford D.; Bellott M.; Dunbrack R. L.; Evanseck J. D.; Field M. J.; Fischer S.; Gao J.; Guo H.; Ha S.; Joseph-McCarthy D.; Kuchnir L.; Kuczera K.; Lau F. T. K.; Mattos C.; Michnick S.; Ngo T.; Nguyen D. T.; Prodhom B.; Reiher W. E.; Roux B.; Schlenkrich M.; Smith J. C.; Stote R.; Straub J.; Watanabe M.; Wiórkiewicz-Kuczera J.; Yin D.; Karplus M. All-atom empirical potential for molecular modeling and dynamics studies of proteins. J. Phys. Chem. B 1998, 102, 3586–3616. 10.1021/jp973084f. 24889800

[ref82] Mackerell A. D.; Feig M.; Brooks C. L. Extending the treatment of backbone energetics in protein force fields: Limitations of gas-phase quantum mechanics in reproducing protein conformational distributions in molecular dynamics simulations. J. Comput. Chem. 2004, 25, 1400–1415. 10.1002/jcc.20065. 15185334

[ref83] Vanommeslaeghe K.; Hatcher E.; Acharya C.; Kundu S.; Zhong S.; Shim J.; Darian E.; Guvench O.; Lopes P.; Vorobyov I.; Mackerell A. D. CHARMM general force field: A force field for drug-like molecules compatible with the CHARMM all-atom additive biological force fields. J. Comput. Chem. 2010, 31, 671–690. 10.1002/jcc.21367. 19575467 PMC2888302

[ref84] Jorgensen W. L.; Chandrasekhar J.; Madura J. D.; Impey R. W.; Klein M. L. Comparison of simple potential functions for simulating liquid water. J. Chem. Phys. 1983, 79, 926–935. 10.1063/1.445869.

[ref85] Humphrey W.; Dalke A.; Schulten K. VMD: Visual molecular dynamics. J. Mol. Graphics Modell. 1996, 14, 33–38. 10.1016/0263-7855(96)00018-5. 8744570

[ref86] Case D. A.; Ben-Shalom I. Y.; Brozell S. R.; Cerutti D. S.; Cheatham T. E. III; Cruzeiro V. W. D.; Darden T. A.; Giambasu G.; Gilson M. K.; Gohlke H.; Goetz A. W.; Harris R.; Izadi S.; Izmailov S. A.; Kasavajhala K.; Kovalenko A.; Krasny R.; Kurtzman T.; Lee T. S.; LeGrand S.; Li P.; Lin C.; Liu J.; Luchko T.; Man V.; Merz K. M.; Miao Y.; Mikhailovskii O.; Monard G.; Nguyen H.; Onufriev A.; Pan S. P.; Qi R.; Roe D. R.; Roitberg A.; Sagui C.; Schott-Verdugo S.; Shen J.; Simmerling C. L.; Skrynnikov J. S.; Swails J.; Walker R. C.; Wang J.; Wilson L.; Wolf R. M.; Wu X.; Xiong Y.; Xue Y.; York D. M.; Kollman D. M. AMBER 2020; University of California: San Francisco, 2020.

[ref87] Grant B. J.; Rodrigues A. P.; ElSawy K. M.; McCammon J. A.; Caves L. S. Bio3d: an R package for the comparative analysis of protein structures. Bioinformatics 2006, 22, 2695–2696. 10.1093/bioinformatics/btl461. 16940322

[ref88] RC Team. R: A Language and Environment for Statistical Computing, 2013.

[ref89] Schrodinger, LLC. The PyMOL Molecular Graphics System, version 2.4, 2020.

[ref90] Bakan A.; Meireles L. M.; Bahar I. ProDy: Protein Dynamics Inferred from Theory and Experiments. Bioinformatics 2011, 27, 1575–1577. 10.1093/bioinformatics/btr168. 21471012 PMC3102222

[ref91] Brooks B. R.; Brooks C. L.; Mackerell A. D.; Nilsson L.; Petrella R. J.; Roux B.; Won Y.; Archontis G.; Bartels C.; Boresch S.; Caflisch A.; Caves L.; Cui Q.; Dinner A. R.; Feig M.; Fischer S.; Gao J.; Hodoscek M.; Im W.; Kuczera K.; Lazaridis T.; Ma J.; Ovchinnikov V.; Paci E.; Pastor R. W.; Post C. B.; Pu J. Z.; Schaefer M.; Tidor B.; Venable R. M.; Woodcock H. L.; Wu X.; Yang W.; York D. M.; Karplus M. CHARMM: The Biomolecular Simulation Program. J. Comput. Chem. 2009, 30, 1545–1614. 10.1002/jcc.21287. 19444816 PMC2810661

[ref92] Schmidt M. W.; Baldridge K. K.; Boatz J. A.; Elbert S. T.; Gordon M. S.; Jensen J. .H.; Koseki S.; Matsunaga N.; Nguyen K. A.; Su S.; Windus T. L.; Dupuis M.; Montgomery J. A. General atomic and molecular electronic structure system. J. Comput. Chem. 1993, 14, 1347–1363. 10.1002/jcc.540141112.

