# Supplementary material for: Catalytic Mechanism of ATP Hydrolysis in the ATPase Domain of Human DNA Topoisomerase IIα
Source: J Chem Inf Model. 2022 Aug 10;62(16):3896–909. doi: 10.1021/acs.jcim.2c00303 (PMC9400105; doi:10.1021/acs.jcim.2c00303)
Supplement: Supplementary file 1 — ci2c00303_si_001.pdf [file ci2c00303_si_001.pdf]

SUPPORTING INFORMATION:

Catalytic Mechanism of ATP Hydrolysis in the ATPase Domain of Human DNA Topoisomerase IIα

Mitja Ogrizek[†1], Matej Janežič[†1], Katja Valjavec[1] and Andrej Perdih[1,2*]

[1] *National Institute of Chemistry, Hajdrihova 19, SI-1001 Ljubljana, Slovenia*

[2] *University of Ljubljana, Faculty of Pharmacy, Aškerčeva 7, SI-1000 Ljubljana, Slovenia*

[†] *These two authors contributed equally to this work.*

Corresponding author:

National Institute of Chemistry

Hajdrihova 19, SI-1001 Ljubljana, Slovenia

E-mail: andrej.perdih@ki.si

Tel.: +386-1-4760-376

1. Movement of the topo IIα ATPase transducer domain during the Molecular Dynamics (MD) simulations

A

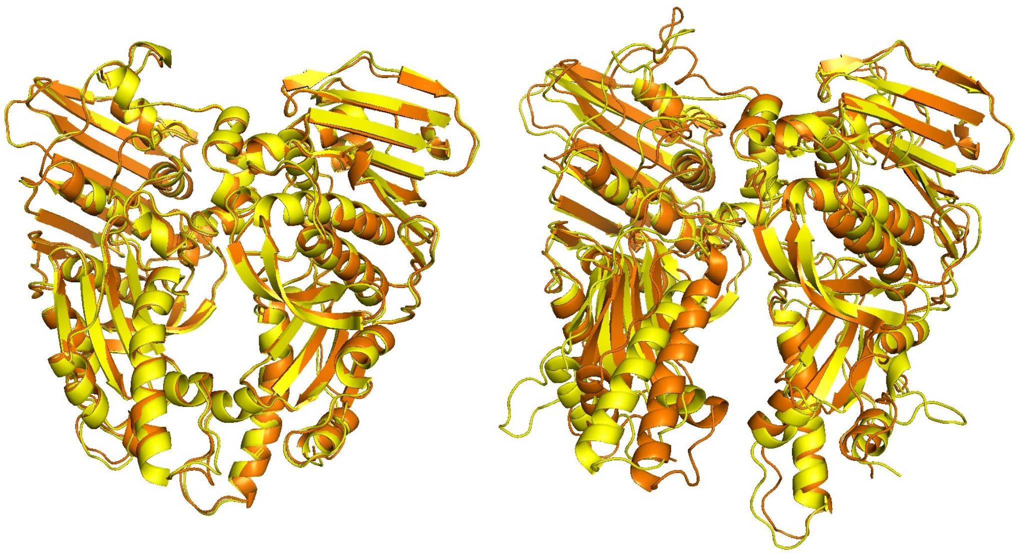

B

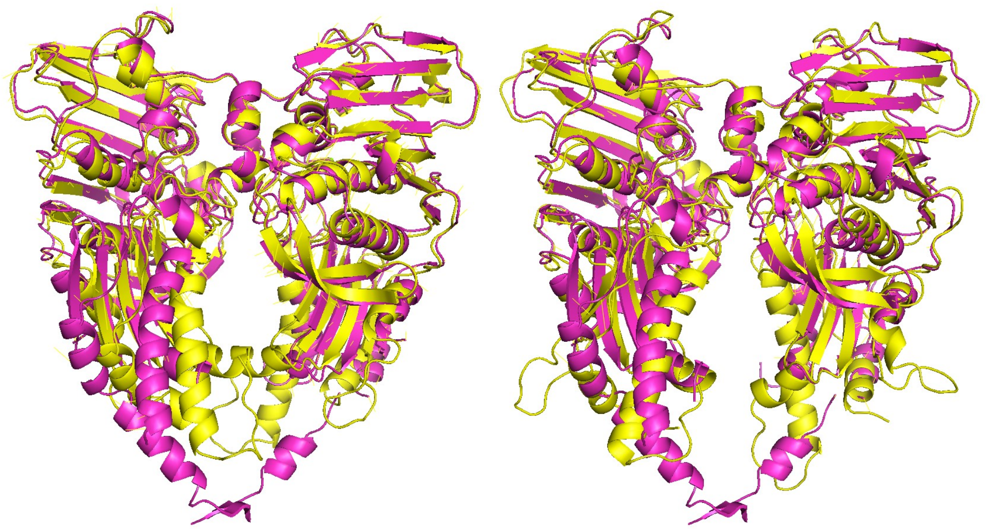

Figure S1. (**A**) Apo system (orange) aligned with holo system (yellow). Left at time 0 ns, right at time 100 ns. Note the bigger opening of the transducer domain in the holo system. (**B**) Holo system (yellow) aligned to pdb 1ZXN (purple, topoisomerase-ADP+SO4 complex). Left at time 0ns, right at time 100ns. Note how the transducer domain in the 100ns structure of the simulated complex more closely aligns to the transducer domain from 1ZXN.

2. Analysis of Molecular Dynamics simulations

A) Comparison of apo and holo systems

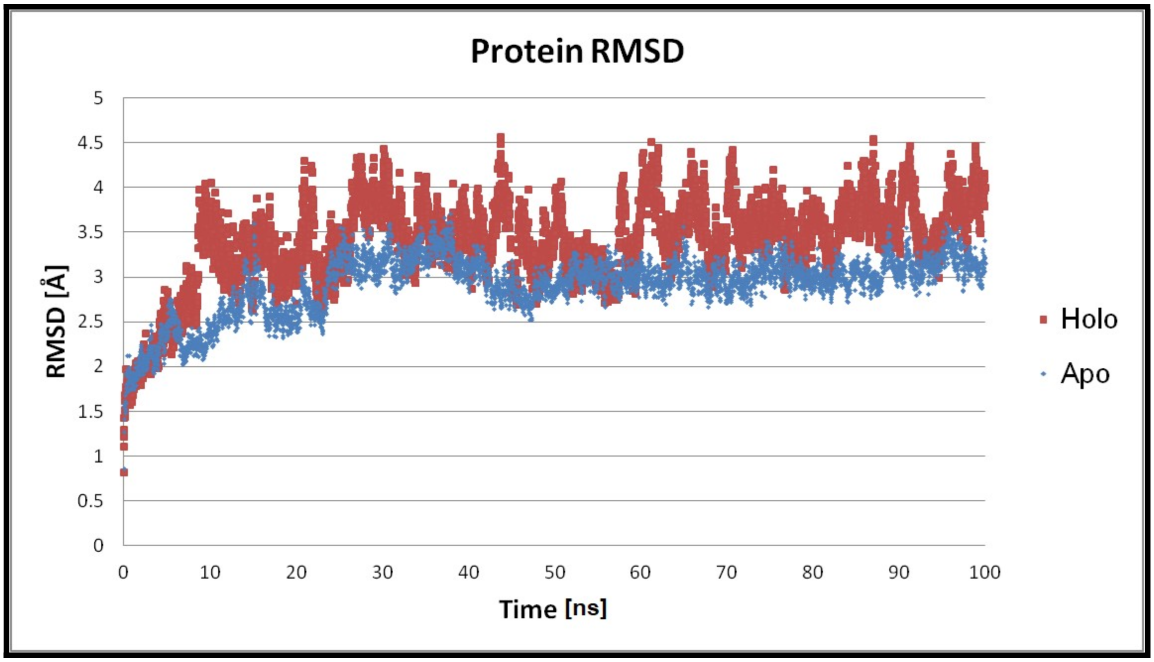

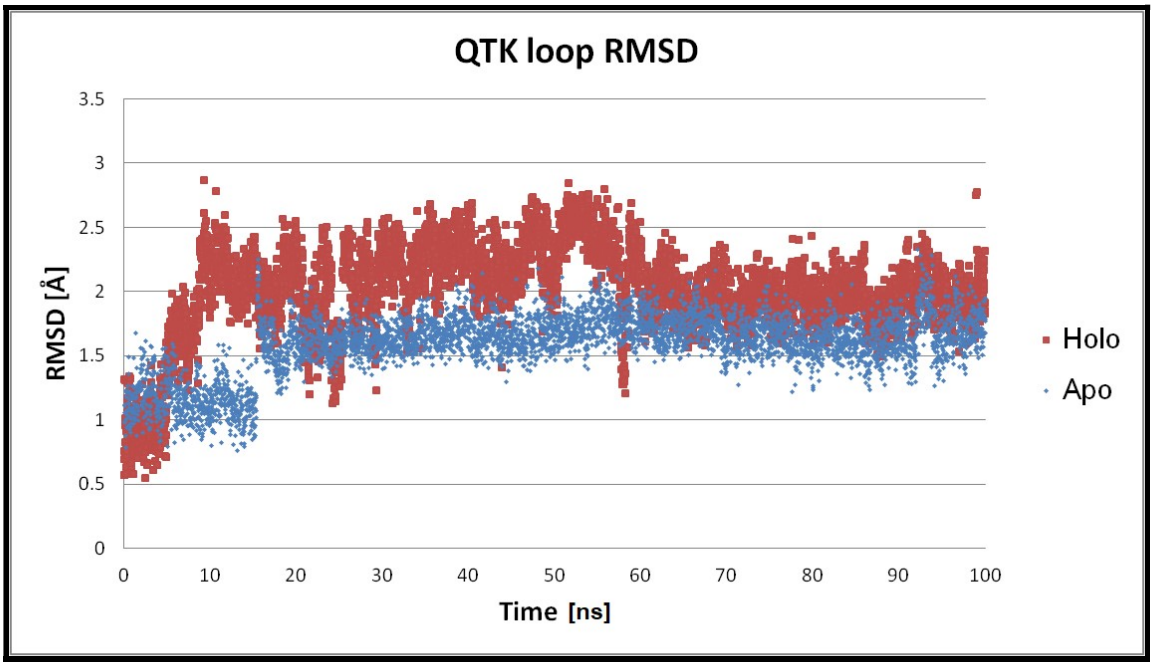

B) Holo system

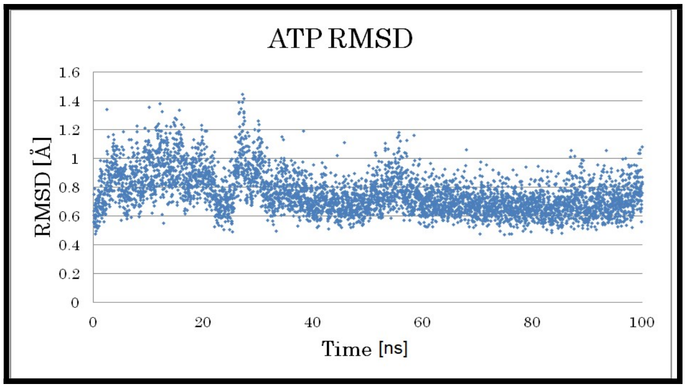

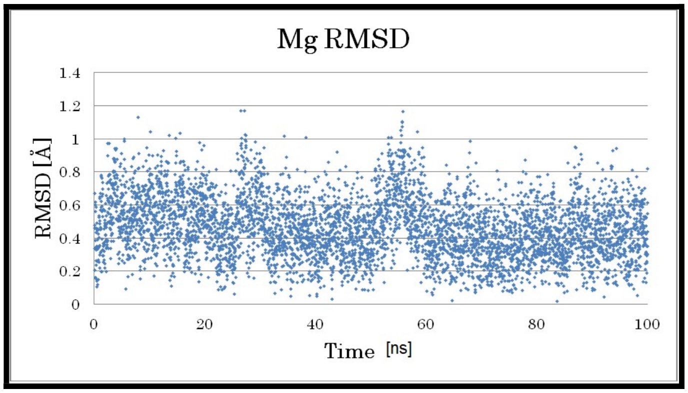

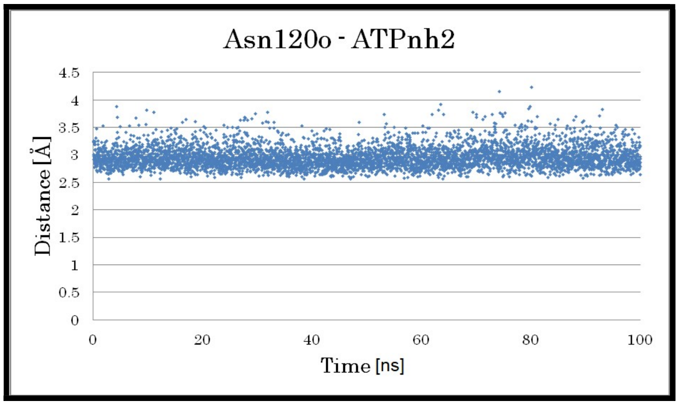

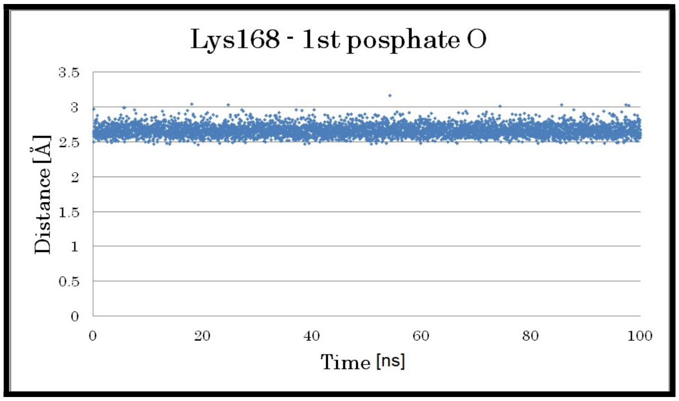

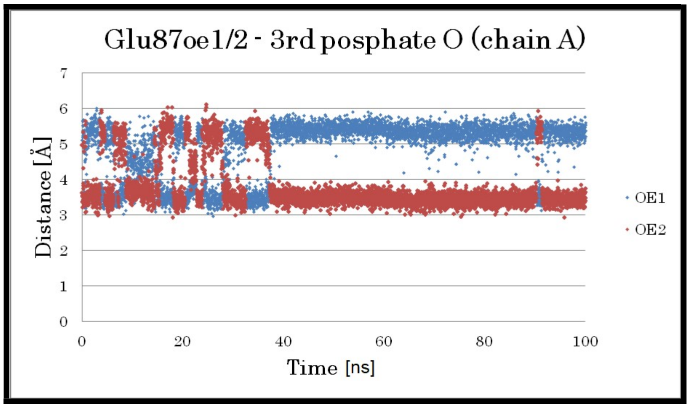

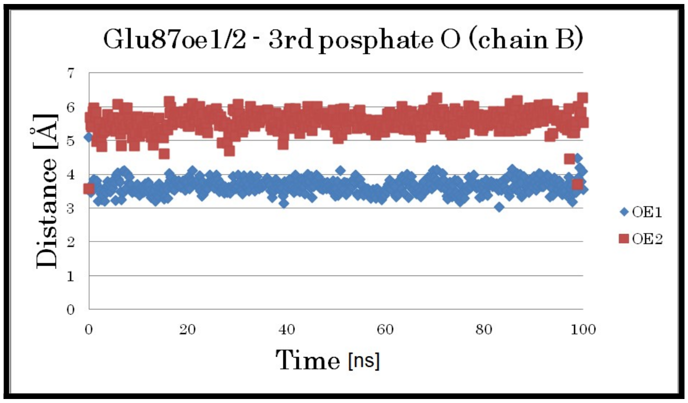

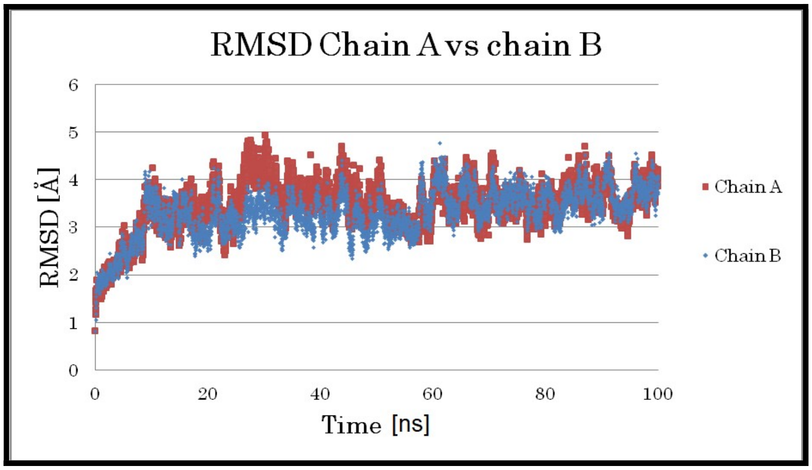

Figure S2. Time-dependent graphs of measured distances during the performed MD simulations for the holo and apo topo IIα systems. For the holo system, unless noted, the graphs are of the distances measured in chain A. Chain B exhibited similar behavior except in the case of Glu87 (see graphs).

Table S1. Average distances between the magnesium ion and the 6 coordinating atoms in the ATPase domain dimer in chain A. Chain B exhibited similar distances.

**Distance**	**[Å]**
Mg^{2+} - α-phosphate	1.90
Mg^{2+} - β-phosphate	1.88
Mg^{2+} -γ-phosphate	1.79
Mg^{2+} -Asn91OD1	2.02
Mg^{2+} - Water 1	2.02
Mg^{2+} - Water 2	1.99

3. Western blot to check for expression of K378 topo IIα mutant

1	**Control**	*10ul*
2	**Control**	*30ul*
3	**K378 A t=2d**	*30ul*
4	**K378 A t=3d**	*30ul*
5	**K378 B t=1d**	*30ul*
6	**K378 B t=2d**	*30ul*
7	**K378 B t=3d**	*30ul*
8	**WT t=3d**	*30ul*
9	**Markers**	
10	**hTii pos control**	*1ul*

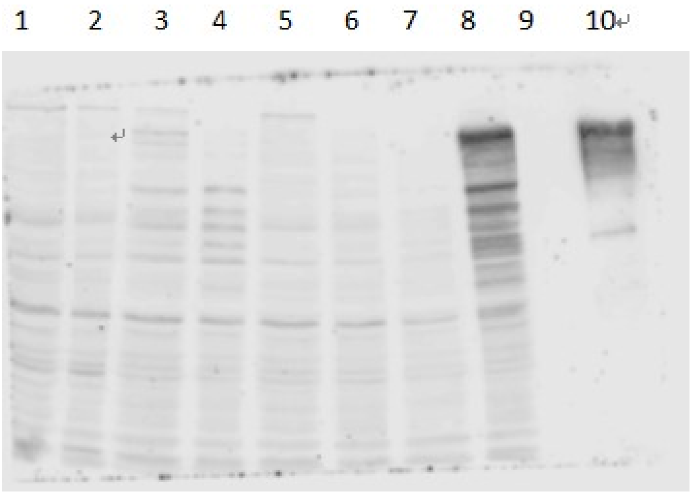

Figure S3. Verification of K378A expression and degradation. The aim was to confirm if the K378 topo IIα mutant is being expressed but degraded. Control cells SF9 were uninfected. K378 A and K378 B were infected on subsequent days with different virus stocks. WT is an infection with the wild type sequence.

4. Results of the ATPase assay

Table S2. ATPase assay results for the WT and mutant K378A human topo IIα; calculated rates, Km (mM ATP) and Vmax (μM ATP/min) values

Average rates for controls without enzyme in the presence of 2 mM ATP (μM/min)				
WT Run 1	WT Run 2	K378 Run 1	K378 Run 2	K378 Run 3
0.412	0.458	0.387	0.388	0.383

Rates (μM/min) at different ATP concentrations (mM) after subtraction of background										
[ATP] mM	2	1.5	1	0.75	0.5	0.25	0.1	0.075	0.05	0.025
WT Run 1	1.120	nd	0.981	0.935	1.222	0.634	0.314	0.118	0.089	-0.023
WT Run 2	0.955	nd	0.880	0.872	0.723	0.580	0.191	0.098	0.051	0.042
K378 Run 1	0.233	nd	0.206	0.192	0.195	0.148	0.102	0.006	0.021	-0.007
K378 Run 2	0.291	0.246	0.264	0.236	0.167	0.186	0.167	0.088	0.085	0.049
K378 Run 3	0.251	0.236	0.183	0.180	0.106	0.067	0.132	0.012	0.085	-0.033

Calculated Km (mM ATP) and Vmax (μM ATP/min) values							
	WT Run 1	WT Run 2	Av. WT	K378 Run 1	K378 Run 2	K378 Run 3	Av. K378
Vmax (μM/min)	1.22	0.96	1.09	0.23	0.29	0.25	0.26
Km (mM)	0.24	0.22	0.23	0.15	0.12	0.096	0.122
kcat (s^{-1})	1.27	1.00	1.14	0.55	0.69	0.6	0.65

nd=not determined

A

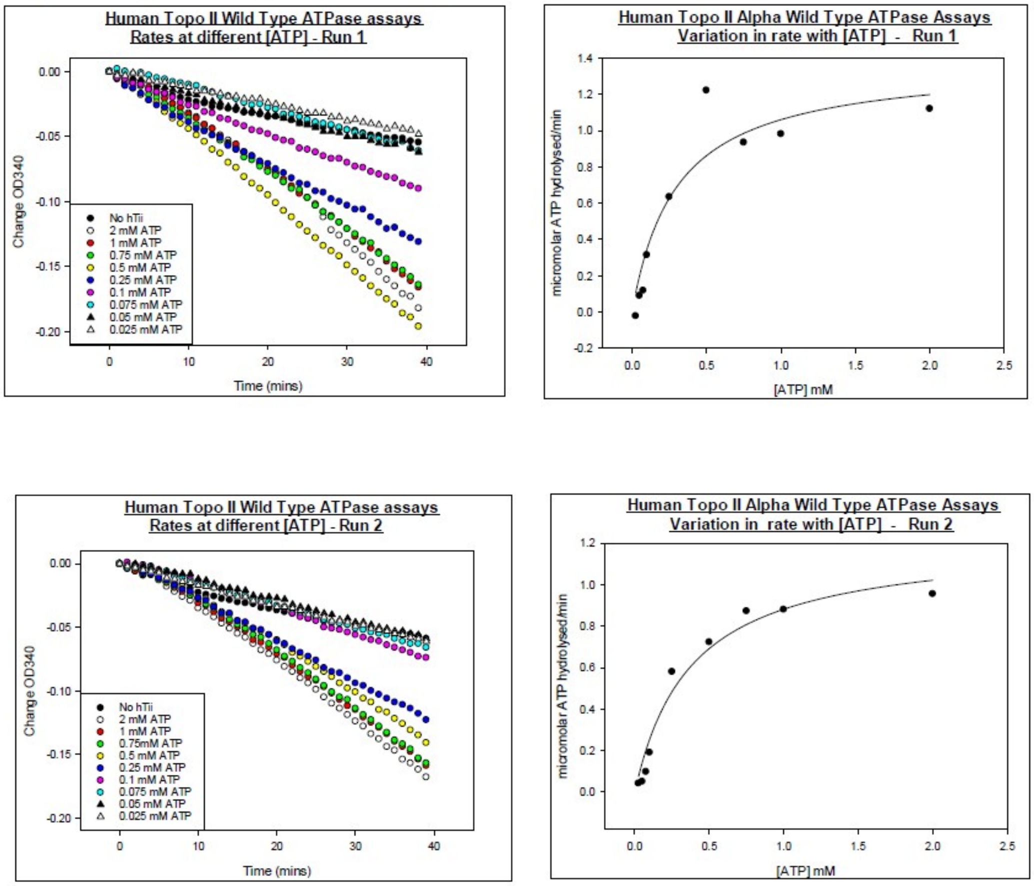
Human Topo II Wild Type ATPase assays
Rates at different [ATP] - Run 1
Change OD340
Time (mins)
No hTii
2 mM ATP
1 mM ATP
0.75 mM ATP
0.5 mM ATP
0.25 mM ATP
0.1 mM ATP
0.075 mM ATP
0.05 mM ATP
0.025 mM ATP
Human Topo II Alpha Wild Type ATPase Assays
Variation in rate with [ATP] - Run 1
micromolar ATP hydrolysed/min
[ATP] mM
Human Topo II Wild Type ATPase assays
Rates at different [ATP] - Run 2
Change OD340
Time (mins)
No hTii
2 mM ATP
1 mM ATP
0.75mM ATP
0.5 mM ATP
0.25 mM ATP
0.1 mM ATP
0.075 mM ATP
0.05 mM ATP
0.025 mM ATP
Human Topo II Alpha Wild Type ATPase Assays
Variation in rate with [ATP] - Run 2
micromolar ATP hydrolysed/min
[ATP] mM

B

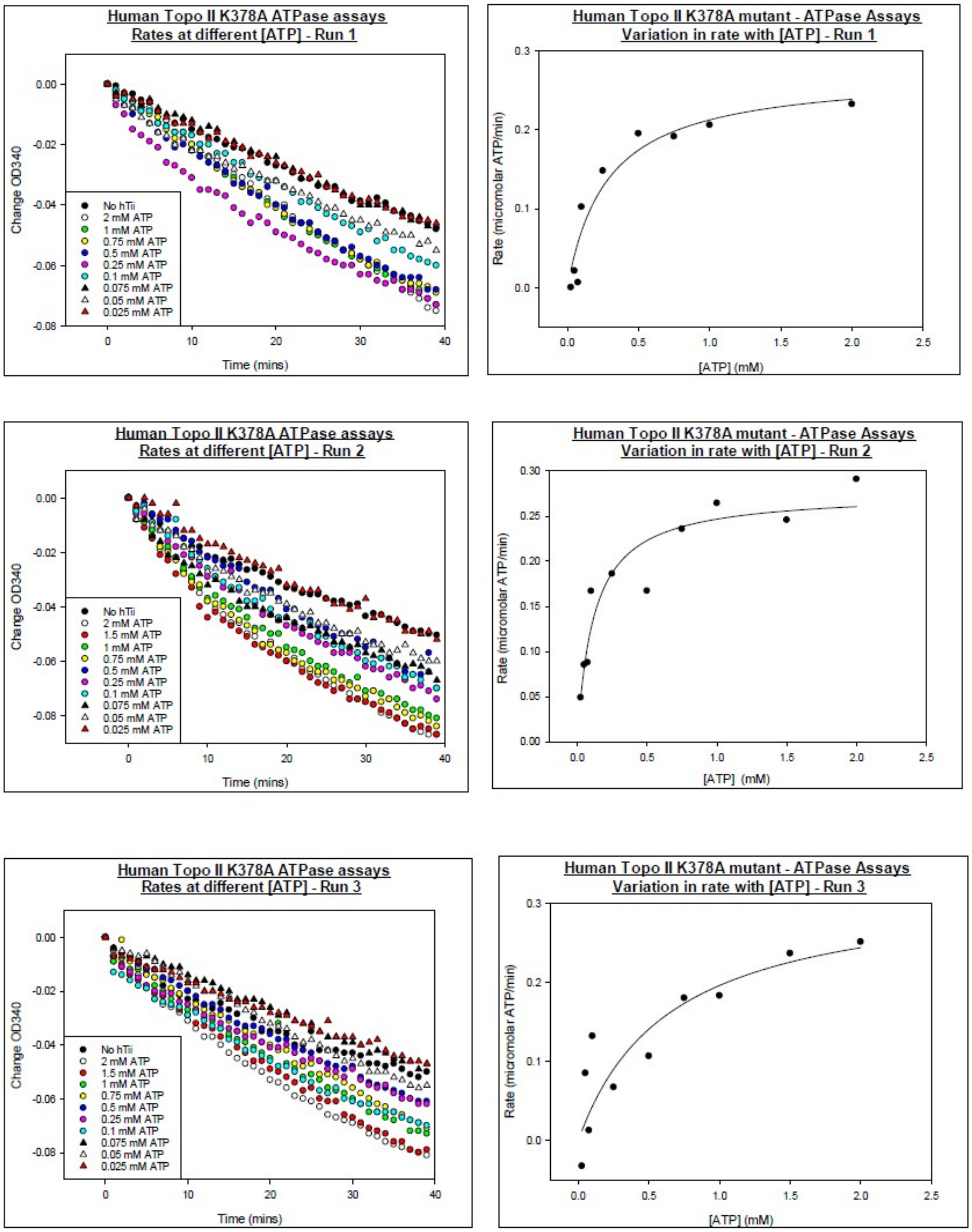

Figure S4. Results of the ATPase assay for A – Wild type and B – K378A human topo IIα mutant. The rates were plotted against ATP concentration and curves fitted using the hyperbolic equation y=ax/(b+x), where y=rate, x= [ATP], a=Vmax, and b=Km.

5. 2D scheme of the QM region

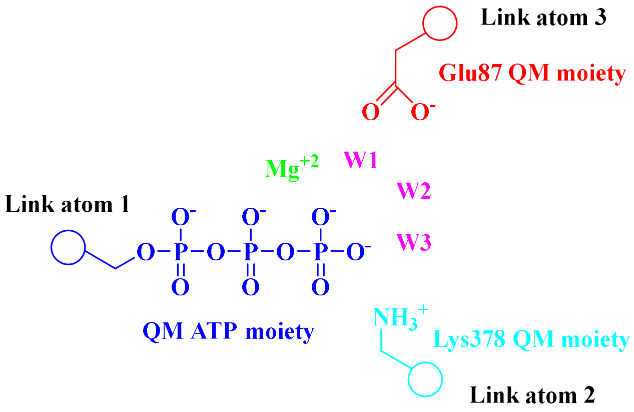

Figure S5. 2D scheme of the selected QM region.
